# Comparative genomic analysis reveals spine formation and coral prostaglandin tolerance in crown-of-thorns starfish

**DOI:** 10.1016/j.fmre.2025.08.016

**Published:** 2025-09-22

**Authors:** Zixuan Liu, Meng Qu, Wanghong Xiao, Geng Qin, Haiyan Yu, Zelin Chen, Shaobo Ma, Xin Wang, Longshan Lin, Qiang Lin

**Affiliations:** a State Key Laboratory of Tropical Oceanography, South China Sea Institute of Oceanology, Guangdong Provincial Key Laboratory of Applied Marine Biology, Chinese Academy of Sciences, Guangzhou 510301, China; b Sanya Institute of Oceanology, SCSIO, Sanya 572000, China; c University of Chinese Academy of Sciences, Beijing 100101, China; d Laboratory of Marine Biology and Ecology, Third Institute of Oceanography, Ministry of Natural Resources, Xiamen 361005, China; e Marine Biodiversity and Ecological EvolutionResearch Center, South China Sea lnstitute of Oceanology, Chinese Academy of Sciences, Guangzhou 510301, China

**Keywords:** Crown-of-thorns starfish, Chromosome evolution, Corallivores, Detoxification, Skeletogenesis

## Abstract

Recurring outbreaks of crown-of-thorns starfish (COTS) are one of the greatest threats to corals and have caused catastrophic damage to coral reef ecosystems in recent decades. As obligate corallivores, COTS can tolerate toxic coral mucus, and the sharp spines covering their bodies provide protection from predators. While both of these characteristics are known to contribute to the vitality of COTS and aggravate the impact of COTS outbreaks, the genetic mechanisms underlying their adaptation remain unclear. In this study, we generated a chromosome-level COTS genome and suggested the importance of chromosome fusion in coral-feeding adaptation. In addition, high expression levels of EP4 receptors in the COTS spine apex indicated their potential ability to use the coral prostaglandin PGE2, which might further activate the skeletogenic gene regulatory network responsible for the growth of the spine and body plan of COTS. A comparison of the historical population sizes of COTS from different locations revealed a unique trend in the Coral Triangle, suggesting a potentially higher risk of outbreaks in this region. Our results provide a molecular basis for the adaptive evolution of COTS and a better understanding of its interactions in coral reef ecosystems.

## Graphical abstract

**Figure:**
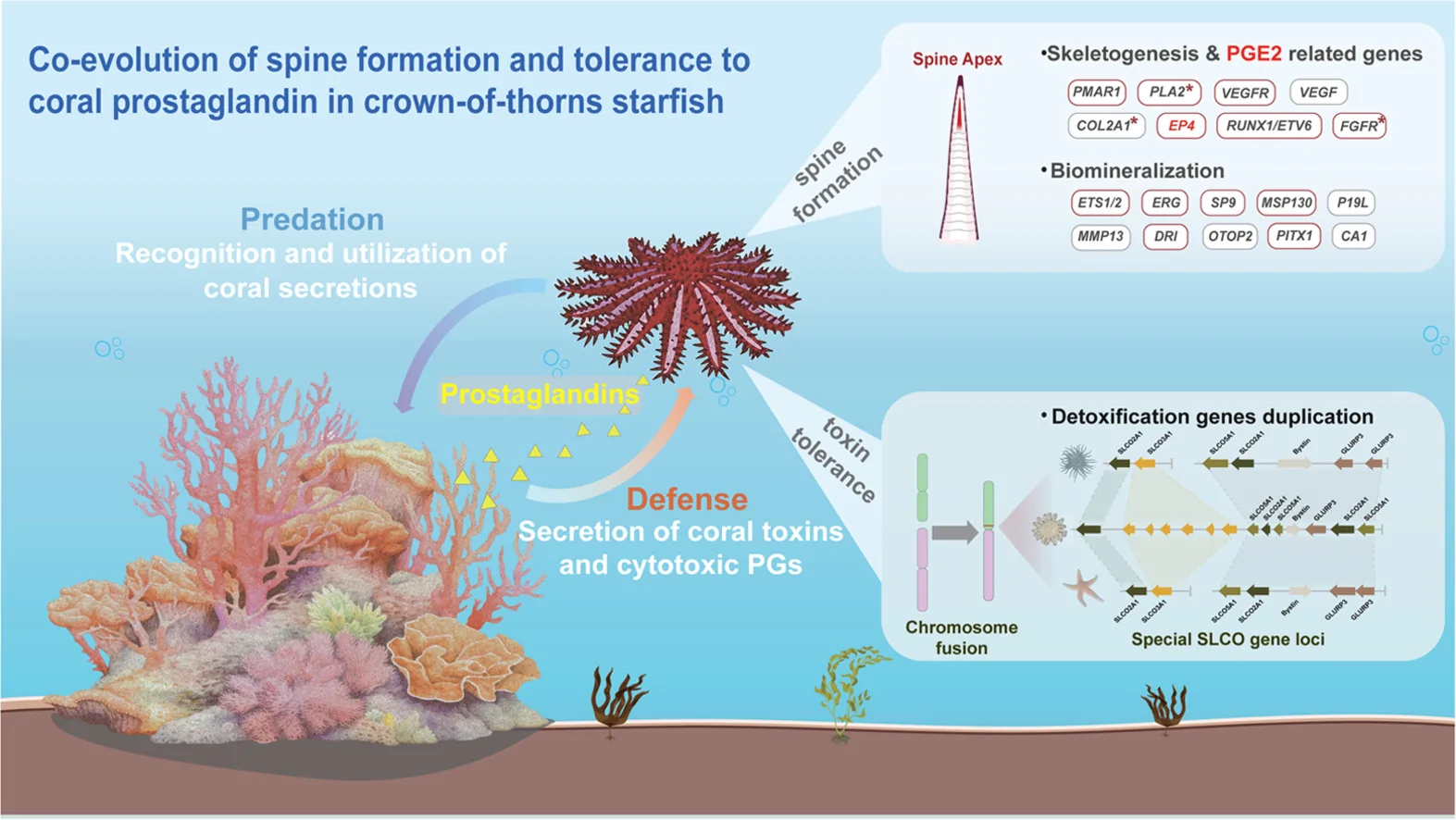

## 1 Introduction

*Acanthaster* spp*.*, often referred to as crown-of-thorns starfish (COTS, excluding *Acanthaster brevispinus*), are the sole venomous starfish and predators of reef-building corals. While COTS generally exist in low densities, population sizes can occasionally experience rapid and substantial growth, termed “outbreaks”, causing significant damage to coral reefs. COTS have significantly contributed to coral reef degradation in the Indo-Pacific region [1]: from 1985 to 2012, the coral cover on the Great Barrier Reef declined from 28.0% to 13.8%, with 48% of this reduction attributed to COTS outbreaks [2]. Furthermore, a heightened frequency of COTS outbreaks has been observed across its entire distribution range in recent years [3]. The fundamental basis for the outbreak of COTS lies in its robust defense mechanisms, including pointed spines that render adult COTS nearly devoid of natural predators [4] and high tolerance to coral toxins, which helps it cope with dietary coral allelochemicals [5]. However, the genetic mechanisms underlying spine formation in COTS and their tolerance to coral toxins remain unclear.

As a ‘well-armored’ species, adult COTS diameters normally range from 60 to 70 cm, with numerous spines covering the upper surface (Fig. 1). The spines consist of calcite ossicles covered by epidermis and dermis [6]. Notably, juvenile COTS are fragile and have yet to develop long hard spines to protect themselves. COTS begin their benthic life as herbivores, with juveniles consuming coralline algae for 13–15 months post-settlement in nature [7]. When they settle, their dietary changes towards becoming corallivores are accompanied by an exponential growth in the size and development of spines, suggesting a strong link between spine formation and coral substances [8]. Recent advances in sea urchin research have revealed that the skeletogenic gene regulatory network (GRN) is vital for calcite endoskeleton formation [9]. This GRN comprises approximately 20 transcription factors and may have been shared by the common ancestor of echinoderms [10]. However, echinoderms are highly diverse in skeletal morphology, implying that the potential regulatory mechanisms of skeletogenesis and biomineralization may differ between species [11]. Thus, there is a need for further research on the genetic mechanisms governing the formation of COTS spines.

**Fig. 1:**
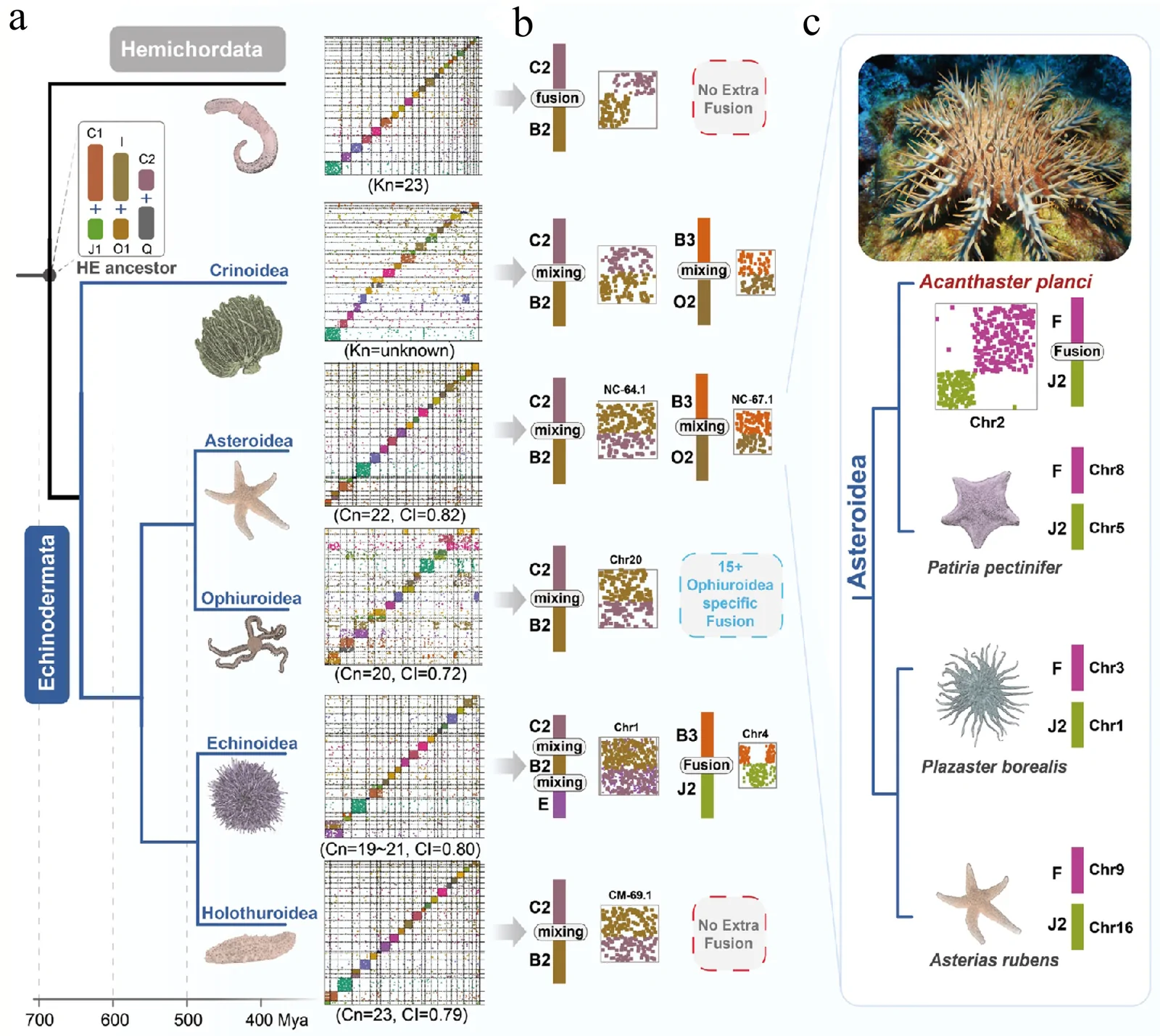
**Oxford dot plot of orthologous genes between BLG and deuterostome.** (a) Phylogenetic relationships of six class species representing hemichordates and echinoderms, and corresponding representative dot plots. CN in parentheses = number of chromosomes in the genome; KN = number of recorded karyotypes. The circle at the node represents the BLG fusion event that existed in the common ancestor of hemichordates and echinoderms (b) and specific fusion or mixing events that occur in each class. (c) A distinctive fusion event in COTS genome, where BLGJ2 and BLGF fused to create the LG02 chromosome. COTS, crown-of-thorns starfish; BLG, bilaterian linkage group.

Corals are among the oldest venomous organisms on the planet [12]. They produce mucus comprising a diverse array of secondary metabolites and allelochemicals. Mucus is vital in fundamental processes such as heterotrophic feeding and sediment purification, while also serving as a defense mechanism that deters predation by fish and other invertebrates [13]. Over the long course of evolution, corallivorous species have evolved counter-adaptive detoxification mechanisms to tackle dietary coral allelochemicals, giving them distinct advantages within their ecosystem [14]. Therefore, understanding the adaptive detoxification mechanisms of COTS is pivotal for understanding the interactions between COTS and corals.

In this study, we assembled the chromosome-level genome of adult COTS (*A. planci*). Using ancestral chromosome reconstruction and comparative genomic analyses, we found that the distinctive chromosomal fusion event observed in the COTS genome resulted in duplication of the prostaglandin transporter gene family, underpinning its specialized adaptations for coral feeding. The coral-derived prostaglandin E2 (PGE2) might promote spine growth and may serve as a crucial signaling factor responsible for releasing COTS from a state of growth “pause”.

## 2 Materials and methods

### 2.1 Animals

COTS samples were collected from the South China Sea (15°52′ N, 115°54′ E). One sample was used for genome sequencing, four for RNA-seq sequencing, and six for behavioral experiments. Samples were dissected onboard and stored at −80 °C until laboratory analysis. All animal experiments were conducted in accordance with our institute’s guidelines and were approved by the Animal Research and Ethics Committee of the South China Sea Institute of Oceanology, Chinese Academy of Sciences (2022–0011). All efforts were made to minimize animal suffering.

### 2.2 Sample preparation and genome sequencing

High-molecular-weight genomic DNA was extracted from the tube feet of the genomic samples using the QIAGEN® Genomic Kit (Cat#13343, Qiagen), according to the standard operating procedure provided by the manufacturer. Paired-end libraries of COTS were generated on the Illumina HiSeq 2000 platform (Illumina), and single-molecule real-time libraries, known as HiFi reads, were sequenced on the PacBio Sequel II platform (PacBio). Hi-C was used to further improve genome assembly, and the obtained sequencing data were constructed using the Illumina Novaseq 6000/MGI-2000 platform at Grandomics (Wuhan, China).

### 2.3 Genome assembly

The de novo assembly of the COTS genome was built with FALCON v0.2.2 (https://github.com/PacificBiosciences/FALCON) using PacBio SMRT reads and polished with NextPolish [15] using Illumina short reads. Quality control of raw Hi-C data was performed using Hi-C-Pro v2.8.1 [16] and low-quality sequences (quality scores < 20), adaptor sequences, and sequences shorter than 30 bp were filtered using Fastp [17]. The chromosome-scale assembly was generated using filtered Hi-C data with LACHESIS (https://github.com/shendurelab/LACHESIS). Completeness of the genome assembly was assessed using BUSCO v4.0.5 [18].

### 2.4 Gene model prediction and annotation

The repeats and transposable elements (TEs) were annotated before gene model prediction using RepeatMasker v4.1.0 (http://www.repeatmasker.org/) and an *ab initio* repeat library for COTS was generated using RepeatModeler v1.1 (http://repeatmasker.org/RepeatModeler.html) and MITE-Hunter (https://github.com/jburnette/MITE-Hunter) (Fig. S2; Table S4). GMATA v2.2 [19] and TRF v4.07b [20] were used to identify simple repeat sequences and all tandem repeat elements within the entire genome. Three independent approaches, *ab initio* prediction, homology search, and transcript assembly, were used for gene prediction in a repeat-masked genome. Finally, EVidenceModeler v1.1.1 (EVM) [21] was used to generate an integrated gene set, while untranslated regions and alternative splicing regions were identified using PASA, based on the RNA-seq assemblies. Gene function information, motifs, and protein domains were assigned by means of comparison with public databases, including SwissProt, NCBI NR (non-redundant), Kyoto Encyclopedia of Genes and Genomes, EuKaryotic Orthologous Groups, and Gene Ontology.

### 2.5 Transcriptome sequencing and analysis

For transcriptome analysis, tissues from the central stomach, pyloric stomach, ampullae, tube feet, aboral spine, aboral spine apex, and hepatic cecum were collected from the three COTS samples. Total RNA was extracted using the TRIzol™ reagent (Molecular Research Center, USA), and paired-end sequencing was performed using the Illumina HiSeq 2000 platform. (Table S9). The raw reads were checked using FastQC v0.11.5 and filtered using Trimmomatic v0.39 [22]. Gene and isoform expression levels were estimated with RSEM v1.3.3 [23], using STAR v2.7.6a [24] as aligners to generate the genome BAM files. STAR-Fusion was used to identify candidate fusion transcripts supported by Illumina reads [25]. Differentially expressed genes were identified using DESeq2. The enrichment analysis of differentially expressed genes was performed using clusterProfiler v3.10 [26].

### 2.6 Gene family and phylogenetic analyses

To elucidate the evolutionary relationship between COTS and other deuterostomes, a comparative genomic analysis was conducted using the genomes of 15 selected species, namely *Chiridota heheva, Parastichopus parvimensis, Plazaster borealis, Strongylocentrotus purpuratus, P. miniata, Hemicentrotus pulcherrimus, Lytechinus variegatus, Saccoglossus kowalevskii, Ptychodera flava, Anneissia japonica, Amphiura filiformis, Ophiura sarsiiLutken, Apostichopus japonicus, Asterias rubens*, and *A. planci*. Ortholog groups (OGs) were determined using OrthoFinder v2.5.4 [27], resulting in 228 single-copy and 3199 one-to-one OGs. Sequence alignments were performed using MAFFT v7.505 [28], with default settings, and trimmed using TrimAl v1.4 [29], with the “-gappyout” option. Sequence alignments were concatenated using FASconCAT-G v1.05 [30] and species trees were constructed using IQ-TREE v2.2.0 [31], with 1000 replicates of ultrafast likelihood bootstrap; the substitution model for sequence alignments was selected using ModelFinder implemented in IQ-TREE. MCMCtree was used to estimate the divergent time based on the concatenated alignments and was calibrated with the fossil record database, as follows: minimum = 371.0 Mya and soft maximum = 463.4 Mya for the first appearance of Holothuroidea; minimum = 89.3 Mya and soft maximum = 145.7 Mya for the first appearance of Echinoidea; minimum = 461.1 Mya and soft maximum = 600.0 Mya for the first appearance of Asteroidea. Gene family expansion and contraction were analyzed using CAFÉ v5 [32] and gene families under *p*-value < 0.05 were used in the subsequent analysis. For specific gene families, the phylogenetic analyses followed a workflow similar to that of the species tree. The divergence of expanded gene families was adjusted for multiple substitutions using the Jukes–Cantor formula *K* = −300/4  ×  Ln (1 − D  ×  4/300). The insertion times were eventually calculated using the formula *T* = *K*/2r [33], in which ‘T’ represents the estimated insertion time and ‘r’ corresponds to the nucleotide substitution rate.

### 2.7 Mutual best hit orthology

Reciprocal BLASTP analysis was conducted on *P. borealis, S. purpuratus, H. pulcherrimus, S. kowalevskii, A. japonica, A. japonicus, A. rubens, A. filiformis*, and *A. planci* against *Branchiostoma floridae*. Mutual best hits were combined to create a set of 6001 core orthologs using an E-value cutoff of 1e–2. We identified boundaries along *B. floridae* chromosomes between blocks of distinct deuterostomes with conserved synteny, by computing the averages of the synteny indicator vector of slide windows containing 20 genes. The positions of these orthologs in the genomes of each species were retrieved using a custom Python script, and the BLGs was constructed based on the amphioxus genome. The shared gene families between the BLGs and species were clustered using Euclidean clustering and finally plotted in R, following the workflow provided in a previous study [34]. To understand the possible evolutionary events in chromosome fusion regions discovered in COTS, further genomic analysis and mapping of syntenic loci between the three starfish genomes were performed using SyMAP v5.4.5 [35].

### 2.8 Identification of positively selected genes

To identify the positively selected genes in the COTS genome, PAL2NAL v14 [36] was used to map codons to protein alignments produced by MAFFT. Positive selection was calculated using the adaptive branch site random-effects likelihood model implemented in HyPhy version 2.3.9 [37]. Each gene was tested by setting COTS as ‘Foreground’. Subsequently, the positively selected gene families were further tested using GWideCodeML v1.1 [38], to ensure the accuracy of the results and obtain positive selection sites within each positively selected gene.

### 2.9 Skeletal development genes

We performed a genome-wide search for skeletogenesis and biomineralization genes in *C. heheva, S. purpuratus*, L. *variegatus, A. japonicus, A. filiformis, O. sarsiiLutken, A. rubens*, and *A. planci*. The structures of these genes differed significantly within three classes of echinoderms. The dataset consists of 31 biomineralization proteins in sea urchins that are important for osteogenesis and 32 skeletogenic genes involved in the skeletogenic GRN in echinoderms [10,39]. However, only 10 homologous biomineralization genes have been identified in the COTS genome. The number of skeletogenic genes is relatively conserved within echinoderms.

### 2.10 Behavioral response of COTS to signals from coral chemicals

COTS were collected from the coral reefs near Wanning, Hainan Province, and transported to the South China Sea Institute of Oceanology, Chinese Academy of Sciences. The COTS were cultured in white fiberglass tanks (3 m × 1.8 m), with continuous 24-hour supply of fresh seawater pumped from nearshore, maintained at a temperature of 26–28 °C, salinity of 38 pp.t, under natural outdoor lighting, and covered with a black shade net on top. Prior to the formal experiments, feeding was withheld for 7 days to induce a state of hunger. To investigate whether COTS can recognize coral secretions, perforated buckets were placed at both ends of the tank, wrapped in white mesh to ensure color consistency with the tank and prevent the COTS from visually detecting the coral. Various Acropora spp. were placed in the experimental group’s buckets, while the control group’s buckets remained empty. Before each experiment, the tanks were thoroughly cleaned, and the water was changed to ensure no residual coral secretions would affect subsequent trials. In each experiment, to ensure the corals had sufficient time to release their secretions, they were left undisturbed in the water for 15 min. During this period, the water pump was turned off to maintain still water, preventing interference with the diffusion of the coral secretions. To avoid aggregation behavior and potential directional bias influencing the results, only one COTS was placed in the tank per trial, and the orientation of each individual was varied between trials. Finally, in each experiment, the movement of the crown-of-thorns starfish was recorded until it reached the stone slab, and the entire process was documented by the DV device (Sony HDR-CX680).

### 2.11 Population demographic history analysis

To infer the historical effective population sizes of the different regions, we conducted a pairwise sequential Markovian coalescence analysis based on paired-end library reads from the SCS sample and nine selected samples from a previous study. Raw data were initially processed using Trimmomatic v0.39, to remove low-quality reads, followed by mapping to the corresponding genome using Bwa v0.7.12. Variant calling for each sample was determined using Bcftools v1.11 (“mpileup” and “call”), an implementation within Samtools v1.11. Substitution mutation rates specific to the four genomes were calculated based on previous research, assuming a generation time of three years. Finally, the estimated population size of each sample was obtained using PSMC v0.6.5 [40] and the plots were manually merged.

## 3 Results and discussion

### 3.1 COTS genome characteristics and chromosomal rearrangements among echinoderms

We sequenced the genome of COTS collected from the South China Sea (SCS) via PacBio HiFi long reads and Illumina paired-end reads, achieving an average coverage depth of 50X (Table S1). The initial assembly displaying high continuity, with a contig N_50_ length of 1.04 Mb (Table S3). By incorporating Hi-C data, we compiled a final assembly of 437 Mb with 21 pseudochromosomes (N_50_ = 21.6 Mb; Fig. S1; Table S2). Genome assessment of COTS using BUSCO showed a high completeness of 97.7% (1.6% fragmented) (Table S2). A total of 16,918 genes were identified through gene prediction, among which 16,431 (97.12%) were supported by transcriptomic evidence. Furthermore, 15,246 genes were successfully annotated based on multiple authoritative databases, including KEGG, NR, KOG, Swiss-Prot, and GO (Table S5). With this high-quality assembly, we proceeded to investigate the evolutionary relationships and genomic features of COTS within the echinoderm lineage.

The phylum Echinodermata is a major group of deuterostomes, with ∼7000 living species found distributed across ocean ecosystems worldwide and have recently become an ecologically important and ideal group for adaptive evolution studies. Using ten available high-contiguity echinoderm genomes and two hemichordate genomes, we reconstruct phylogenetic relationships and ancestral linkage groups across major echinoderms lineages (Table S6). The results of the maximum-likelihood phylogenetic tree and molecular clock analysis based on these genomes are in accordance with previous studies (Figs. S3, S4) [41]. We then constructed Bilaterian linkage groups (BLGs) based on Reciprocal Best Hit orthologous genes between the amphioxus and selected genomes, and identified 23 BLGs (Fig. S5). Three fusion events (BLGJ1-BLGC1, BLGQ-BLGC2, and BLGI-BLGO1) were conserved in both hemichordates and echinoderms, reflecting the shared BLG characteristics inherited from their common ancestors (Fig. 1a). A comparison between hemichordates and echinoderms showed that the transition of BLG B2 and C2 from fusion to mixing may have occurred in the common ancestor of the echinoderm, a finding recently corroborated by the brittle star genome [42].

Most echinoderm chromosomes maintained their original BLG structure and had a 1:1 correspondence with the amphioxus chromosomes (Figs. S5-S7). This conserved synteny between echinoderms and amphioxus may be attributed to low rates of genomic rearrangement and selection for retaining linkages between genes and their neighboring regulatory elements [43]. Each class of echinoderms exhibits unique and conserved BLG fusion or mixing events within their genomes, and species-specific fusion events are allowed in different classes of echinoderms (Fig. 1b). These additional BLG fusions or mixing events could physically link genes on different chromosomes and form a recombinant gene pattern that could facilitate their evolution and speciation. Notably, COTS displayed a distinctive fusion event, in which BLGJ2 and BLGF were fused to create the LG02 chromosome (Fig. 1c). Macrosynteny showed that these two sets of genes remain localized within their respective segments, rather than undergoing redistribution across the length of the fused chromosome, as seen in ancient fusion events [44], suggesting that it belongs to a recent fusion event that might be related to the speciation and adaptive evolution of COTS.

### 3.2 Fragment duplication facilitates gene duplications related to novel traits in COTS

Chromosome fusions, a major type of chromosomal rearrangement, play a significant role in genome evolution and species divergence [45]. Recent studies have demonstrated that chromosomal fusions contribute to the formation of adaptive genomic clusters, facilitate adaptation to local environments, and often form co-opted and novel traits during long-term evolution [46,47]. To explore adaptive loci in the COTS fusion chromosome, we identified syntenic regions between COTS and two starfish species (*A. rubens* and *P. borealis*) via SyMAP, revealing extensive rearrangements within the fused chromosome (Figs. 2a, and S8, S9). Remarkably, the duplication of chromosome fragments was found near the fusion zone, leading to the expansion of prostaglandin transporters (*SLCO2A1, SLCO3A1, SLCO5A1*; Fig. 2b).

**Fig. 2:**
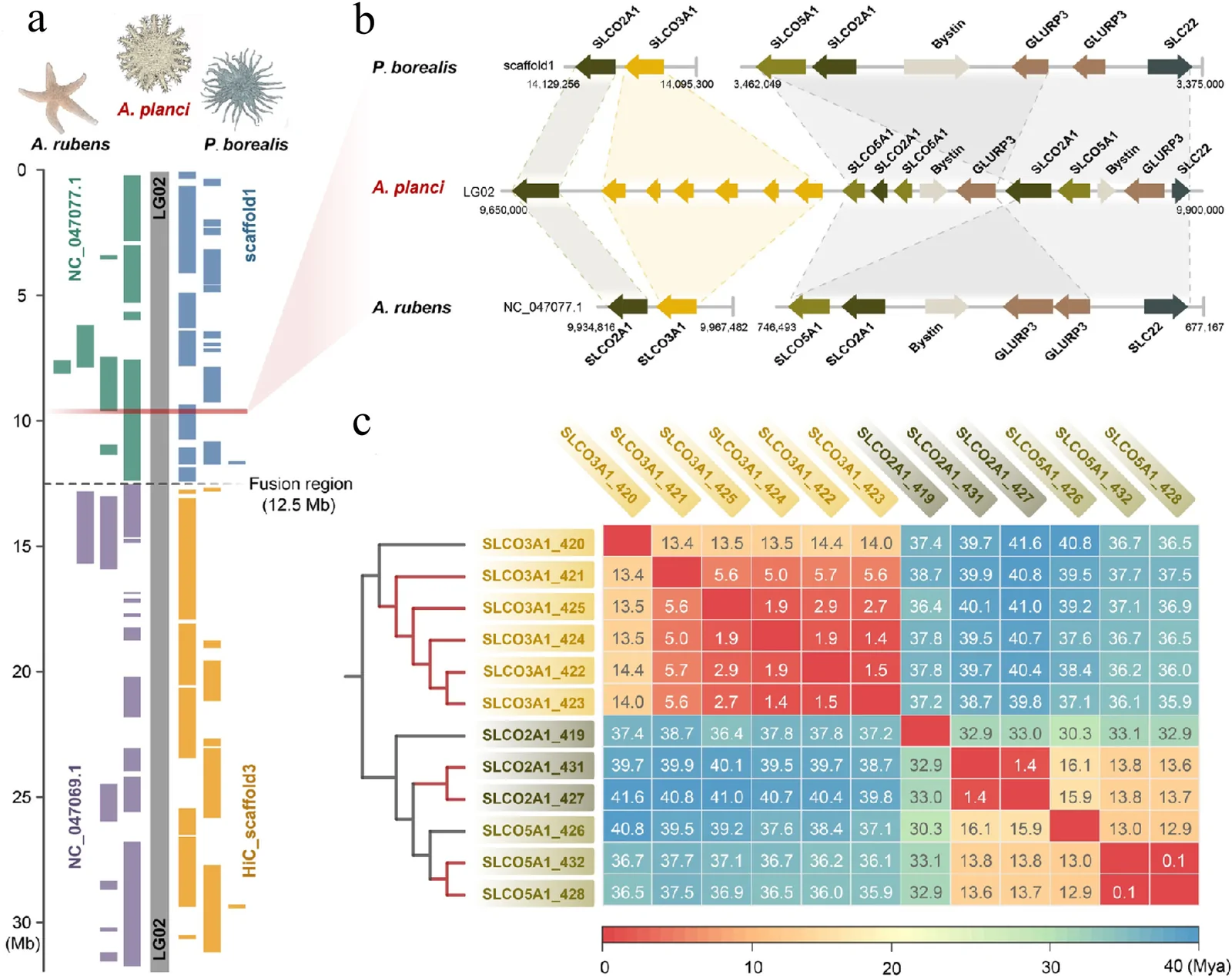
**Syntenic structure of fused chromosome and adaptive linkage cluster.** (a) Syntenic map between the fused chromosome from COTS and two chromosomes from *Plazaster borealis* and *Asterias rubens*. The colored rectangles on either side of chromosomes are chromosomal fragmentation maps, while the red line represents the location of the adaptive loci. (b) Syntenic map of adaptive loci between COTS and the two other reference species. The colored rectangles represent two different gene loci. (c) Estimated insertion time of expanded *SLCO* gene families in the adaptive loci. The red line in the phylogenetic tree indicates that these genes are newly formed by means of chromosome fusion. COTS, crown-of-thorns starfish.

The duplication of these genes occurred approximately 1–5 Mya (Fig. 2c), a time period when coral populations in the ‘Coral Triangle’ began to flourish [48]. *SLCO2A1* and *SLCO5A1* have been reported to facilitate the cellular uptake of prostaglandins (PGs) and regulate both their local and systemic distribution, especially those of PGE2, which plays an important role in the PG signaling pathway [49]. Since corals secrete PGs as anti-predatory agents [50], the chromosome fusion-induced duplication likely enhanced COTS detoxification of coral mucus, providing a selective advantage. Similar adaptive benefits are observed in three-spined sticklebacks, where chromosomal fusions drive phenotypic divergence, including variations in armor plating, body shape, and feeding style, contributing to adaptive divergence in traits and adaptation to the local environments [46].

In COTS, we found high expression levels of *SLCO2A1* and *SLCO5A1* in the stomach and hepatic cecum (the digestive glands of starfish) (Fig. S13), paralleled by upregulated expression of *HPGD* (encoding 15-hydroxyprostaglandin dehydrogenase; Fig. 3a) [51], a pivotal enzyme involved in PG degradation. This co-expression pattern implies that COTS may possess the ability to utilize dietary PGs from corals and selectively degrade anti-predatory PGs. These findings suggest that COTS chromosome fusions and rearrangements may underpin its unique ability to tolerate and process PG-rich coral mucus, supporting its corallivorous niche.

**Fig. 3:**
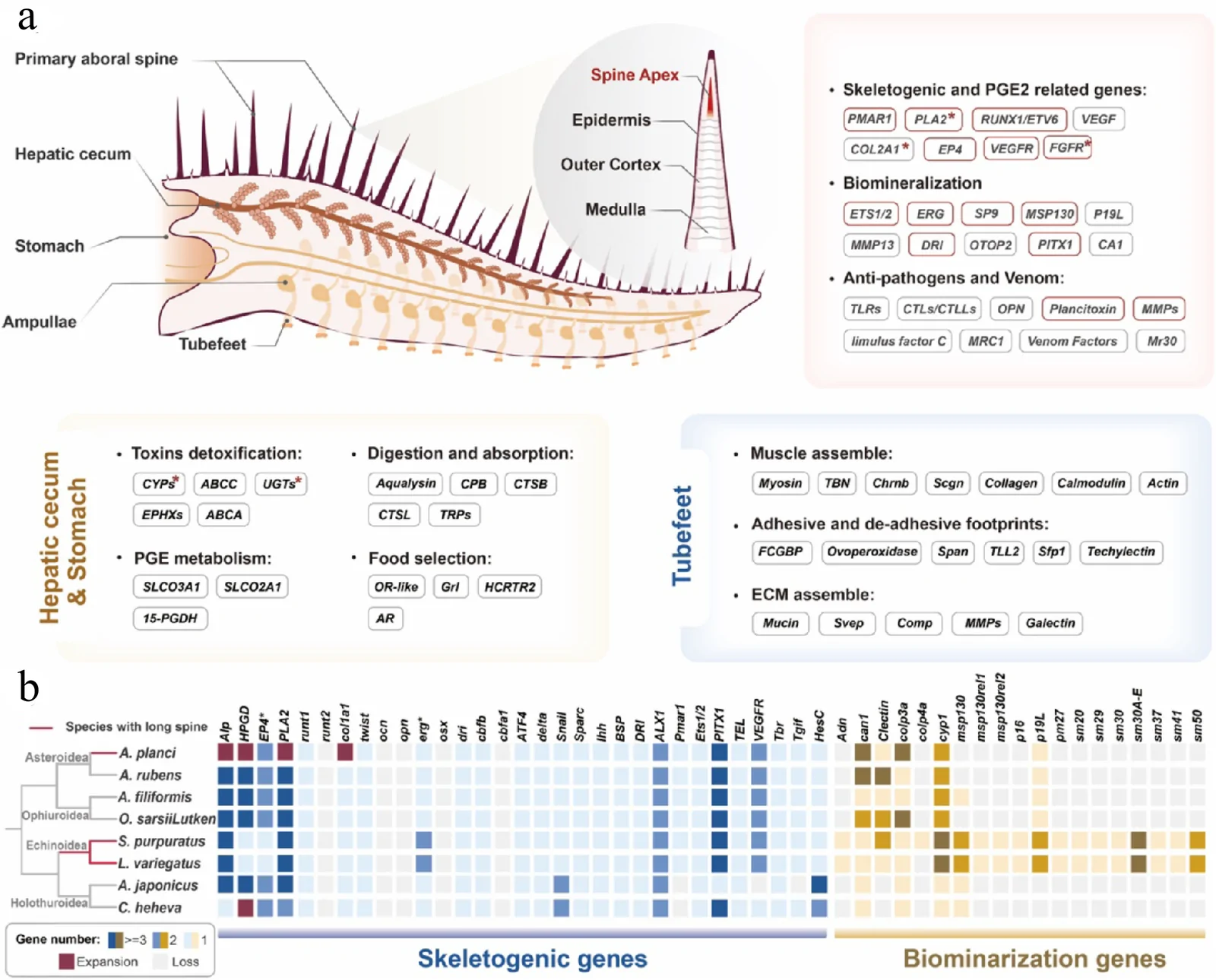
**Expression and structure of skeletogenic genes in crown-of-thorns starfish.** (a) Correlation between high expression genes and corresponding characteristics in each tissue. The asterisk indicates that the gene is undergoing positive selection or expansion. The genes highlighted in red are those that are highly expressed only at the apex of the spine. (b) Structure of biomineralization and skeletogenic genes in selected echinoderms. Most of the genes involved in spine formation in sea urchins are missing in starfish.

### 3.3 Genetic and evolutionary signatures of coral toxin tolerance in COTS

Corals are an allelochemically rich source, producing chemicals, such as palytoxins, PGs, acetogenins, prostanoids, and highly functionalized steroids, all of which exhibit significant anti-predator activity [52]. To explore the genetic basis of toxin tolerance, we identified 40 gene families that exhibited expansions in the COTS genome (Table S7), enriched in metabolic pathways, specifically xenobiotic metabolism (Fig. S10). Among these, Cytochrome P450 (*CYP*), glutathione S-transferase (*GST*), very long-chain acyl-CoA synthetase, and solute carrier family 6 member 5 (*SLC6A5*; Table S7) have been implicated in the metabolism of toxic xenobiotics in marine mollusks [53,54]. The expansion of these metabolic genes in the COTS genome underlines their potential role in the adaptation to coral toxins.

After millions of years of predation on corals, many corallivorous species have evolved allelochemical tolerance mechanisms to retain access to food resources that are mechanically and/or chemically defended. In the case of the butterfly fish (*Chaetodon multicinctus*), the primary solution is to convert dietary allelochemicals into less toxic metabolites through genes that participate in the detoxification of diet-derived compounds, such as *CYPs, GST*, epoxide hydrolase (*EPHX*), and UDP-glucuronosyltransferase (*UGT*) [14]. In COTS, the *GST* and *CYP* gene families have expanded significantly, with 20 and 7 copies, respectively, compared to an average of 10 and 3 copies in other Asteroidea species. Interestingly, we also found that one copy each of *UGT* and *CYP* was positively selected for COTS (Table S8). Similarly, in florivorous insects, the co-option and duplication of CYPs have also been found, which enhance their tolerance to plant defensive chemicals [55], suggesting that this is the result of natural selection and highlighting the importance of coral allelochemicals in driving the evolution of COTS. Detoxification pathways are typically divided into three phases: Phase I enzymatic reactions modify compounds by adding or exposing polar atoms (*CYPs*), Phase II reactions increase polarity by conjugating Phase I products with polar molecules (*UGT*), and Phase III involves the excretion or compartmentalization of metabolites using transmembrane pumps (ABC transporters, *ABCs*). Transcriptomic analysis revealed that *CYPs, UGTs, EPHXs*, and *ABCs* were highly expressed in COTS’s stomach and hepatic cecum, suggesting their role in the detoxification of dietary coral toxins (Figs. 3a and S14). Over millions of years of evolution, COTS have developed sophisticated detoxification mechanisms to exploit coral resources despite their chemical defenses, providing them with ecological advantages in the coral reef ecosystems.

### 3.4 Spine formation is related to skeletogenic gene expression patterns in COTS

Normally, juvenile COTS transition to coral prey after 13–15 months of consuming crustose coralline algae and then mature at 2–3 years in nature [3,7]. Previous studies show they can remain in a herbivorous “growth pause” state (16–18 mm diameter) for up to 6.5 years, with stunted body size and spine development until coral prey is available, indicating that coral-related substances may exert a direct influence on the skeletogenesis of COTS (Fig. 4d). To understand the potential mechanisms of skeleton formation in COTS, we searched for the classic skeletogenic GRN, which is well-defined in echinoderms [56], in the COTS genome. This GRN comprises *ets1/2, tel, tbr, alx1, hex, vegfr, fgfr, pitx1*, and *erg*, which are involved in cell migration, cell proliferation, biomineralization, and epithelial-to-mesenchymal transition (EMT) [57]. Although the GRN is largely conserved, evolutionary differences among echinoderms allow for special skeletal morphology and formation mechanisms [11]. Indeed, the genome-wide search for genes involved in biomineralization and skeletogenesis showed that compared with “spine-equipped” sea urchins, COTS have a significantly different gene number (Fig. 3b). Unlike sea urchins, which rely on unique genes (*SM30–50, p16, pm27*, and *Msp130-related*) for spine formation, COTS lack these genes but exhibit positive selection in *erg, fgfr*, and *EP4* (Table S8). The transcription factor *erg* (ETS family) regulates skeletogenic phenotypes and cell migration, while fibroblast growth factor receptor (*fgfr*) is essential for skeletal formation and regeneration in echinoderms [10]. Furthermore, the signaling receptor EP4, which serves as a prostanoid receptor for PGE2, participates in PGE2-dependent cellular growth and bone formation in mammals, primarily *via* the cyclic adenosine monophosphate (cAMP)-protein kinase A (PKA) and phosphoinositide 3-kinase (PI3K)-Akt signaling pathways [58]. Notably, extracellular tyrosine-protein kinase PKDCC (*PKDCC*) and citrate transporter solute carrier family 13-member 5 (*SLC13A5*) gene families, key regulators of vertebrate mineralization, have undergone significant expansion in COTS (Figs. S11, S12) [59,60]. Additionally, transcript fusions in cell proliferation genes (especially *OXTR, MIB1*, and *mTOR*) were widespread across tissues (Fig. S15), potentially enhancing growth regulation and contributing to unique spine morphology [61]. Thus, transcript fusions in COTS may also be involved in growth regulation, and these subtle variations in gene expression patterns may be associated with their unique spine structures. Together, these genomic features may reflect a distinctive regulatory architecture potentially contributing to COTS skeletogenesis and its specialized skeletal morphology as corallivores.

### 3.5 Insights into a potential role of coral PGE2 and EP4 signaling in COTS skeletogenesis

The horizontal structure of COTS spine (primary aboral spine) can be classified, from outer to inner, into three components: epidermis, outer cortex (dermis), and medulla composed of ossicles [62], with the growth of the spine persisting at the apex (Fig. 3a). Therefore, for the first time, we sampled both the apex and base of aboral spines to reconstruct gene expression patterns of their skeletons as accurately as possible. Compared with other tissues and the two stages of COTS samples (juvenile and brachiolaria), the aboral spine apex had 721 highly expressed genes, which were largely associated with the mitogen-activated protein kinase (MAPK), Ras, cAMP-PKA, and PI3K-Akt signaling pathways (Fig. 4a). The MAPK and Ras signaling pathways regulate the migration of primary mesenchyme cells (PMCs) and biomineralization [63], and can be activated by fibroblast and vascular endothelial growth factor receptors, which are also highly expressed in the aboral spine apex (Fig. 4c). Besides, the highly expressed genes in the aboral spine apex also included those related to the GRN (*pitx1, mmp13, ets1/2, tel, dri*, and *erg*) and skeletogenesis (*runx1, msp130, hox11/13b, wnt9–10*, and *p19l*) (Fig. 4c), highlighting that the spine apex is the critical site for adult COTS skeleton formation.

**Fig. 4:**
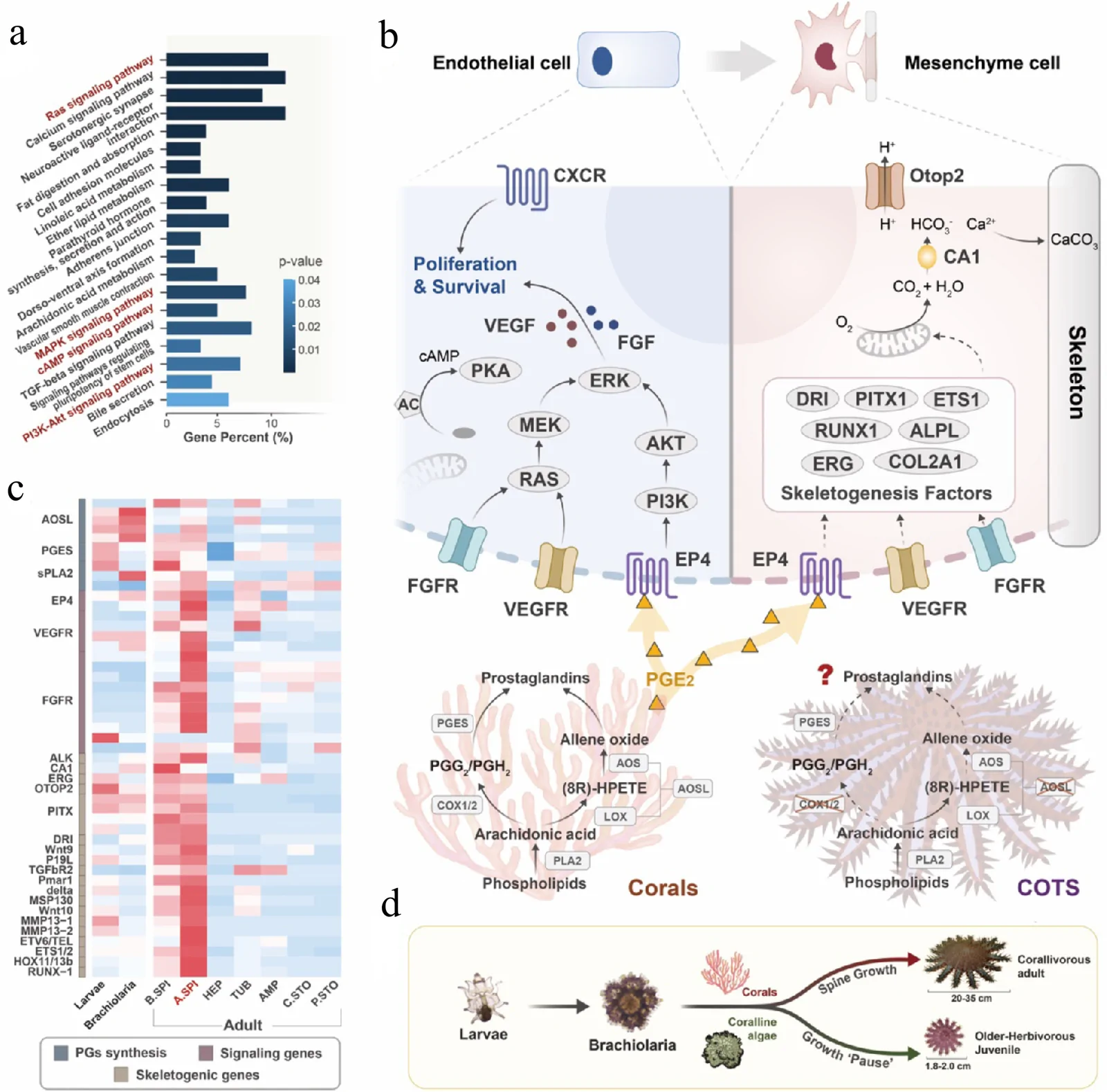
**Mechanism of skeleton formation in the spine apex, and the role of coral mucus on the growth of spines.** (a) Kyoto Encyclopedia of Genes and Genomes enrichment result of genes that were highly expressed in the spine apex. (b) Schematic representation of the mechanism by which coral PGE2 stimulates skeleton formation and elongation of the COTS spine. Two possible prostaglandin synthesis pathways appear to be missing in COTS. (c) Expression profiles of skeletogenic genes in different tissues and stages of COTS. Several conserved genes involved in primary mesenchymal cells, gene regulatory network, and epithelial-to-mesenchymal transition are highly expressed in the spine apex. (d) The influence of dietary shifts (coral-derived PGE2) on the life cycle of COTS. COTS, crown-of-thorns starfish.

Notably, the elevated expression of *EP4* (PGE2 receptor EP4 subtype) in the spine apex suggests exogenous PGE2 stimulation, as COTS and other echinoderms lack key prostaglandin synthesis genes (*COX1/2*). In vertebrates and corals, PGE2 is produced by prostaglandin E synthase (PGES) from the prostaglandin endoperoxide intermediates PGG2 and PGH2. These intermediates are derived from arachidonic acid *via* the (11R)-cyclooxygenase (*COX*) pathway [64,65]. Corals can also produce cyclopentenone prostaglandins *via* the (8R)-lipoxygenase pathway mediated by allene oxide synthase/ lipoxygenase (*AOSL*) (Fig. 4b) [65], while echinoderms retain *AOSL* but lack the allene oxide synthase region (Fig. S16). This suggests that these genes in echinoderms may only exhibit lipoxygenase activity. We speculate that the function of allene oxide synthase may be performed by other genes, such as *cyp74A* in plants, which future studies may confirm. The minimal expression of PGE2 synthesis genes (*AOSL, PGES* and secretory phospholipase A2 (*sPLA2*)) in the spine apex further supports that EP4 signaling likely responds to exogenous PGE2, as observed in other marine invertebrates accumulating environmental PGs [66,67]. Concurrently, high expression of defense/immune-related neuropeptides and secretory enzymes in COTS spine indicates that the spine is not only simple defensive body surface appendage but also a potential sensory organ [68], supported by COTS’ behavioral responses to *Acropora* spp. chemicals (Fig. S19). These results suggest that COTS may possess the capability to recognize and utilize coral secretions *via* its spines, which include substances conducive to spine growth, such as PGE2.

In sea urchins, the initial calcite endoskeleton is built by PMCs and skeletogenic cells produced *via* EMT, which regulate skeletal morphology during the early larval stage. PMCs disappear before metamorphosis, while secondary mesenchyme cells (SMCs) emerge and have the potential to translate into skeletogenic cells [69]. Although starfish lack PMCs, they possess mesenchymal cells that share similarities with the SMCs of sea urchins that migrate from the tip of the archenteron [69]. In COTS, we found a significant expansion of the gene encoding the Ras-related protein Rab-24 (*Rab24*; five copies *vs.* one in other starfish; Table S7), a conserved regulator of autophagy, protein trafficking, and EMT [70]. The expansion of *Rab24* indicates the importance of EMT in the evolution of COTS. The high expression of key genes involved in PMC gene regulatory network and EMT (*Pmar1, erg, tel, ets1/2*, and *dri*) in the aboral spine apex (Fig. 4c), suggests that its skeletogenic cells originate from EMT-derived mesenchymal cells (Fig. 4b). Additionally, *otop2l*, a conserved biomineralization gene previously thought to be specific to sea urchin larval PMCs [71], exhibited high expression levels in both larvae and adult COTS, further supporting the presence of functional mesenchymal cells in adult spines. These findings indicate that mesenchymal cells in adult COTS spines may mediate spine elongation and skeleton formation in response to coral-derived PGE2.

### 3.6 Heavy outbreak history/way of COTS in the coral triangle

On the western border of the Coral Triangle Region, the South China Sea (SCS) provides a favorable habitat for COTS populations (Fig. 5a). From 2018 to 2019, an outbreak led to an increase in COTS density on the Paracel Islands from 400 individuals per hectare to 1000 individuals per hectare [72], with a similar outbreak on the Spratly Islands, leading to significant coral cover decline in the SCS. A previous study reconstructed the demographic history of COTS in seven regions [73]. In this study, we complemented this analysis by investigating historical changes in the population size of COTS in the SCS (Figs. 5b and S17, S18). The results showed that the first fluctuation in COTS abundance occurred during the Mid-Pleistocene Transition (MPT, 0.7–1.25 million years ago) (Fig. 5b), a period marked by dramatic climatic changes, including enhanced glaciation and a drop in sea levels that exposed shelf breaks and upper-slope deposits. During the MPT, the population size of *Acropora* corals, the preferred food source for COTS, also increased, suggesting that fluctuations in COTS abundance may be linked to food resource availability [74]. After the mid-Brunhes Event (MBE), a climatic transition similar to the MPT, the COTS population in the SCS continued to increase until approximately 0.2 Mya, while populations in other regions declined. This period coincided with the highest primary productivity observed in borehole sediment samples from the SCS (Site ODP 1143), reflected by the lowest percentage of the coccolithophore *Florisphaera profunda*, a widely used productivity proxy (Fig. 5b) [75]. The highest primary productivity following MBE may be attributed to the simultaneous intensification of winter and summer monsoons, resulting in shallower nitraclines and enhanced uptake of terrigenous nutrient salts from the land [76]. The stronger East Asian monsoon likely created more favorable conditions for COTS larvae, sustaining population growth in the SCS while other regions experienced declines [77]. More importantly, the SCS is now even more coral-diverse than the center of the ‘Coral Triangle Region’ [78], implying that the SCS could probably face a greater threat of COTS outbreaks in the future.

**Fig. 5:**
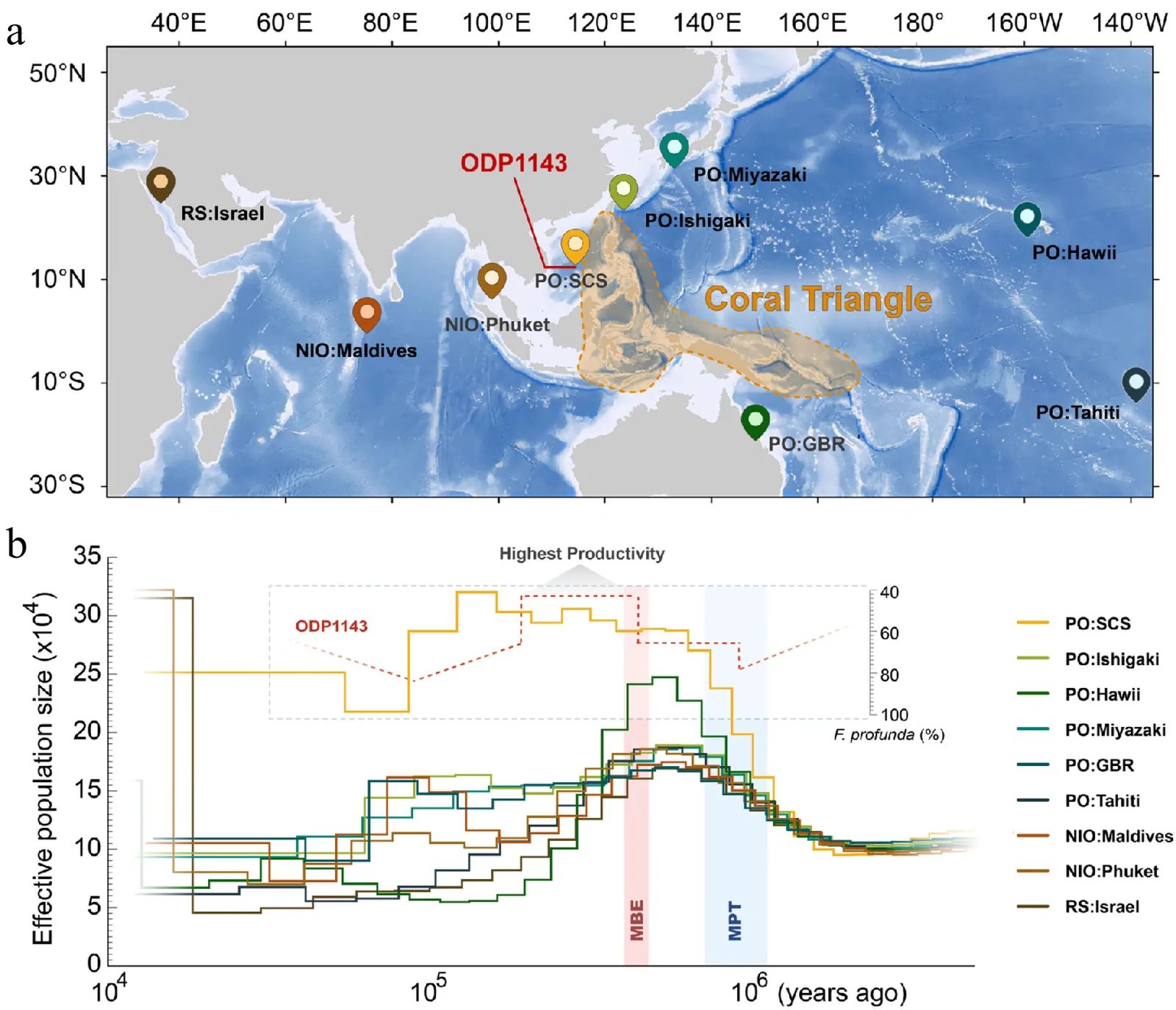
**Population demographic history of COTS in eight regions.** (a) The actual locations of the eight regions are marked on the map. (b) Effective population sizes of COTS were inferred using the pairwise sequential Markovian coalescence method, with a generation time of 3 years and mutation rates of 9.9e-9, 9.4e-9, and 9.8e-9 bp per generation in the NIO, and RS, and PO species, respectively. The mutation rates were assumed from the estimated genome size, referring to previous studies. Blue shade during 0.7–1.25 Mya: MPT; Red shade during 0.4–0.44 Mya: MBE. The red dotted line represents the change in the proportion of Florisphaera profunda. NIO, Northern Indian Ocean; RS, Red Sea; PO, Pacific Ocean; MPT, Mid-Pleistocene transition; MBE, Mid-Brunhes event; COTS, crown-of-thorns starfish.

## 4 Conclusion

In this study, we present a high-quality COTS genome and explore potential evolutionary mechanisms that may contribute to adaptation to corallivory. Comparative genomic analyses have provided evidence that chromosome fusion may have played a key role in the speciation of COTS by forming special gene loci that may facilitate the utilization of coral mucus by COTS and their tolerance towards it. Our integrative multiomic analyses revealed gene expression patterns in the spines of COTS, suggesting the significance of the spine apex in the processes of skeletogenesis and coral mucus recognition, thereby providing a better understanding of the formation of the skeleton in echinoderms. We additionally reconstructed the historical population size of the South China Sea (SCS) population, which enriched our understanding of the evolutionary history of COTS. Overall, our chromosome-level COTS genome is a valuable asset for advancing echinoderm research and provides new perspectives for preventing COTS outbreaks.

## Data accessibility

The COTS genome project has been deposited in NCBI under the accession number PRJNA1039947. Raw sequencing data of the transcriptomes were deposited in NCBI under the accession number SRR25848494-SRR26848519. The genome assembly and related annotation files can be accessed through Figshare at 10.6084/m9.figshare.24571057.

## CRediT authorship contribution statement

**Zixuan Liu:** Writing – review & editing, Writing – original draft, Visualization, Software, Formal analysis. **Meng Qu:** Writing – review & editing, Writing – original draft, Visualization, Conceptualization. **Wanghong Xiao:** Investigation. **Geng Qin:** Methodology. **Haiyan Yu:** Software, Formal analysis. **Zelin Chen:** Software, Methodology. **Shaobo Ma:** Data curation. **Xin Wang:** Software, Methodology. **Longshan Lin:** Writing – review & editing, Supervision. **Qiang Lin:** Writing – review & editing, Supervision, Project administration, Methodology.

## Declaration of competing interest

The authors declare that they have no conflicts of interest in this work.

## Appendix Supplementary materials

Supplementary information: Supplementary Document S1 includes Full methods, Figures S1–S19, and Table S1–9. Image, application 1
