# Supplementary material for: Comparative genomic analysis reveals spine formation and coral prostaglandin tolerance in crown-of-thorns starfish
Source: Fundam Res. 2025 Sep 22;6(5):3056–65. doi: 10.1016/j.fmre.2025.08.016 (PMC13644484; doi:10.1016/j.fmre.2025.08.016)
Supplement: Supplementary file 1 [file mmc1.pdf]

Supplementary information

Comparative Genomic Analysis Reveals Spine Formation and Coral Prostaglandin Tolerance in Crown-of-Thorns Starfish

This Supplementary Material includes:

1. Full Methods
2. Supplementary Fig S1 to S19
3. Supplementary Table S1 to S9
4. References

Full Methods

Sample preparation and genome sequencing

COTS samples were collected from the SCS (15°52'N, 115°54'E) and stored in liquid nitrogen until they reached the laboratory in Guangzhou. High-molecular weight genomic DNA was extracted from the tube feet of the samples using the QIAGEN® Genomic Kit (Cat#13343, Qiagen), according to the standard operating procedure provided by the manufacturer. Paired-end libraries of COTS were generated on the Illumina HiSeq 2000 platform (Illumina), and single-molecule real-time libraries, known as HiFi reads, were sequenced on the PacBio Sequel II platform (PacBio). Hi-C was used to further improve genome assembly, and the obtained sequencing data were constructed using the Illumina Novaseq 6000/MGI-2000 platform at Grandomics (Wuhan, China). The Illumina platform yielded 228,924,674 clean reads, providing an average sequencing depth of 50.06×. The PacBio platform generated 690,334 HiFi reads, with an average depth of 18.16×.

Genome assembly

Raw sequencing data generated using PacBio SMRT were processed using the quality control protocol through the SMRT link v8.0, to remove low-quality reads and adapters, resulting in subreads. After obtaining the subreads, the *de novo* assembled genome was assembled using FALCON v0.2.2 (https://github.com/PacificBiosciences/FALCON). All PacBio SMRT reads were then mapped back to the contigs with BLASR[1] and pbgcpp v2.0.0 was used to correct sequencing errors with default parameters, according to the alignments. The contigs were refined with NextPolish[2] using Illumina short reads, with the default parameters. To discard possibly redundant contigs and generate a final assembly, similarity searches were performed with the parameters "identity 0.8 – overlap 0.8". Quality control of raw Hi-C data was performed using Hi-C-Pro v2.8.1[3], as previously described. First, low-quality sequences (quality scores<20), adaptor sequences, and sequences shorter than 30 bp were filtered using Fastp[4], following which the clean paired-end reads were mapped to the draft assembled sequence using Bowtie2 v2.3.2[5] (-end-to-end --very-sensitive -L 30), to obtain unique mapped paired-end reads. Valid interaction-paired reads were identified from uniquely mapped paired-end reads and retained using HiC-Pro, for further analysis. The scaffolds were further clustered, ordered, and oriented into scaffolds onto chromosomes using LACHESIS (https://github.com/shendurelab/LACHESIS). The completeness of the genome assembly was assessed using BUSCO v4.0.5 with the metazoa_odb10 database[6]. To evaluate the accuracy of the final assembly, all Illumina PE reads were mapped to the assembled genome using bwa v0.7.12[7] and the mapping rate and genome coverage of the sequencing reads were assessed using Samtools v1.11[8].

Gene model prediction and annotation

The annotation process was initiated by detecting tandem repeats using GMATA v2.2[9] and TRF v4.07b[10]. GMATA was used to identify simple repeat sequences and TRF was used to recognize all tandem repeat elements within the entire genome. To identify the transposable elements (TEs) in the COTS genome, we used a combination of *ab initio*- and homology-based methods. Initially, we used MITE-Hunter (https://github.com/jburnette/MITE-Hunter) and RepeatModeler v1.1[11] to predict an *ab initio* repeat library for COTS, with default settings. Subsequently, we aligned this library with TEclass Repbase[12], to classify the type of each repeat family. To further identify repeats across the entire genome, we applied RepeatMasker v4.1.0 (http://www.repeatmasker.org/) to search for both known and

novel TEs, by aligning sequences against the *de novo* repeat and Repbase TE libraries. The overlapping TEs belonging to the same repeat class were finally consolidated and merged.

Three independent approaches, *ab initio* prediction, homology search, and reference-guided transcriptome assembly, were used for gene prediction in a repeat-masked genome. For *de novo* prediction, RNA sequencing (RNA-seq) reads were assembled *de novo* using Stringtie and analyzed using the PASA pipeline v2.3.3 to produce a training set. GeMoMa v1.6.1[13] was used to align the homologous peptides from related species to the assembly and obtain the gene structure information, which was used for homolog prediction. Finally, for RNA-seq-based gene prediction, filtered mRNA sequencing reads were aligned to the reference genome using STAR-2.7.3a[14], with default settings. Finally, EVidenceModeler v1.1.1 (EVM)[15] was used to generate an integrated gene set, while untranslated regions and alternative splicing regions were identified using PASA, based on the RNA-seq assemblies. The longest transcripts for each locus were retained, and regions outside the open reading frames were designated as untranslated regions. Gene function information, motifs, and protein domains were assigned by means of comparison with public databases, including SwissProt, NCBI NR (non-redundant), Kyoto Encyclopedia of Genes and Genomes, EuKaryotic Orthologous Groups, and Gene Ontology. Putative domains and Gene Ontology terms of the genes were identified using the InterProScan v5.59-5.91[16], with default parameters. For the other four databases, BLASTP was used to compare the EVM-integrated protein sequences against the four well-known public protein databases, with an E-value cutoff of 1e−05, and the hits with the lowest E-value were retained. The results of the five database searches were finally concatenated.

Transcriptome sequencing and analysis

For transcriptome analysis, tissues from the central stomach, pyloric stomach, ampullae, tube feet, aboral spine, aboral spine apex, and hepatic cecum were collected from the three COTS samples. Total RNA was extracted using the TRIzol™ reagent (Molecular Research Center, USA), and paired-end sequencing was performed using the Illumina HiSeq 2000 platform. The raw reads were checked using FastQC v0.11.5 and filtered using Trimmomatic v0.39[17]. Gene and isoform expression levels were estimated with RSEM v1.3.3[18], using STAR v2.7.6a[14] as aligners to generate the genome BAM files. Differentially expressed genes were identified using DESeq2. Only genes that were overexpressed with a fold-change above 2 and false discovery rate below 0.05 against other tissue types were classified as highly expressed, and their corresponding enrichment analysis was performed using clusterProfiler 3.10[19].

Gene family and phylogenetic analyses

To elucidate the evolutionary relationship between COTS and other deuterostomes, a comparative genomic analysis was conducted using the genomes of 15 selected species, namely *Chiridota heheva*, *Parastichopus parvimensis*, *Plazaster borealis*, *Strongylocentrotus purpuratus*, *Patiria miniata*, *Hemicentrotus pulcherrimus*, *Lytechinus variegatus*, *Saccoglossus kowalevskii*, *Ptychodera flava*, *Anneissia japonica*, *Apostichopus japonicus*, *Amphiura filiformis*, *Ophiura sarsii*, *Asterias rubens*, and *Acanthaster planci,* Ortholog groups (OGs) were determined using OrthoFinder v2.5.4[20], resulting in 228 single-copy and 3199 one-to-one OGs. Sequence alignments were performed using MAFFT v7.505[21], with default settings, and trimmed using TrimAl v1.4[22], with the "-gappyout" option. Sequence alignments were concatenated using FASconCAT-G v1.05[23] and species trees were constructed using IQ-TREE v2.2.0[24], with 1000 replicates of ultrafast likelihood bootstrap; the

substitution model for sequence alignments was selected using ModelFinder implemented in IQ-TREE. MCMCTree was used to estimate the divergent time based on the concatenated alignments and was calibrated with the fossil record database, as follows: minimum=371.0 Mya and soft maximum=463.4 Mya for the first appearance of Holothuroidea; minimum=89.3 Mya and soft maximum=145.7 Mya for the first appearance of Echinoidea; minimum=461.1 Mya and soft maximum=600.0 Mya for the first appearance of Asteroidea. Gene family expansion and contraction were analyzed using CAFÉ v5[25] and gene families under p-value<0.05 were be used in the subsequent analysis. For specific gene families, the phylogenetic analyses followed a workflow similar to that of the species tree. The divergence of expanded gene families was adjusted for multiple substitutions using the Jukes–Cantor formula $K=-300/4 \times \mathrm{Ln}\ (1 - D \times 4/300)$. The insertion times were eventually calculated using the formula $T=K/2r$[26], in which 'T' represents the estimated insertion time and 'r' corresponds to the nucleotide substitution rate.

Mutual best hit orthology

Reciprocal BLASTP analysis was conducted on *P. borealis*, *S. purpuratus*, *H. pulcherrimus*, *S. kowalevskii*, *A. japonica*, *A. japonicus*, *A. rubens*, and *A. planci* against *Branchiostoma floridae*. Mutual best hits were combined to create a set of 6001 core orthologs using an E-value cutoff of 1e–2. We identified boundaries along *B. floridae* chromosomes between blocks of distinct deuterostomes with conserved synteny, by computing the averages of the synteny indicator vector of slide windows containing 20 genes. The positions of these orthologs in the genomes of each species were retrieved using a custom Python script, and the BLGs was constructed based on the amphioxus genome. The shared gene families between the BLGs and species were clustered using Euclidean clustering and finally plotted in R, following the workflow provided in a previous study[27]. After identifying the syntenic relationships between chromosomes of each species and the BLGs, we manually annotated the BLG fusion and mixing events for each echinoderm species based on the following criteria. "Fusion" refers to the relatively simple end-to-end joining of ancestral linkage groups, typically indicating a more recent evolutionary event and considered to be a reversible process. In contrast, "mixing" describes a more complex structure that involves extensive internal rearrangements and reshuffling of genes from different ancestral chromosomes following fusion[28]. To understand the possible evolutionary events in chromosome fusion regions discovered in COTS, further genomic analysis and mapping of syntenic loci between the three starfish genomes were performed using SyMAP v5.4.5[29].

Identification of positively selected genes

To identify the positively selected genes in the COTS genome, PAL2NAL v14[30] was used to map codons to protein alignments produced by MAFFT. Positive selection was calculated using the adaptive branch site random-effects likelihood model implemented in HyPhy version 2.3.9[31]. Each gene was tested by setting COTS as 'Foreground'. Subsequently, the positively selected gene families were further tested using GWideCodeML v1.1[32], to ensure the accuracy of the results and obtain positive selection sites within each positively selected gene.

Skeletal development genes

We performed a genome-wide search for skeletogenesis and biomineralization genes in *C. heheva*, *S. purpuratus*, *L. variegatus*, *A. japonicus*, *A. filiformis, O. sarsii, A. rubens*, and *A. planci*. The structures of these genes differed significantly within three classes of echinoderms. The dataset consists of 31

biomineralization proteins in sea urchins that are important for osteogenesis and 32 skeletogenic genes involved in the skeletogenic GRN in echinoderms[33,34]. However, only 10 homologous biomineralization genes have been identified in the COTS genome. The number of skeletogenic genes is relatively conserved within echinoderms.

Population demographic history analysis

To infer the historical effective population sizes of the different regions, we conducted a pairwise sequential Markovian coalescence analysis based on paired-end library reads from the SCS sample and nine selected samples from a previous study. Raw data were initially processed using Trimmomatic v0.39, to remove low-quality reads, followed by mapping to the corresponding genome using Bwa v0.7.12. Variant calling for each sample was determined using Bcftools v1.11 ("mpileup" and "call"), an implementation within Samtools v1.11. Substitution mutation rates specific to the four genomes were calculated based on previous research, assuming a generation time of three years. Finally, the estimated population size of each sample was obtained using PSMC v0.6.5[35] and the plots were manually merged.

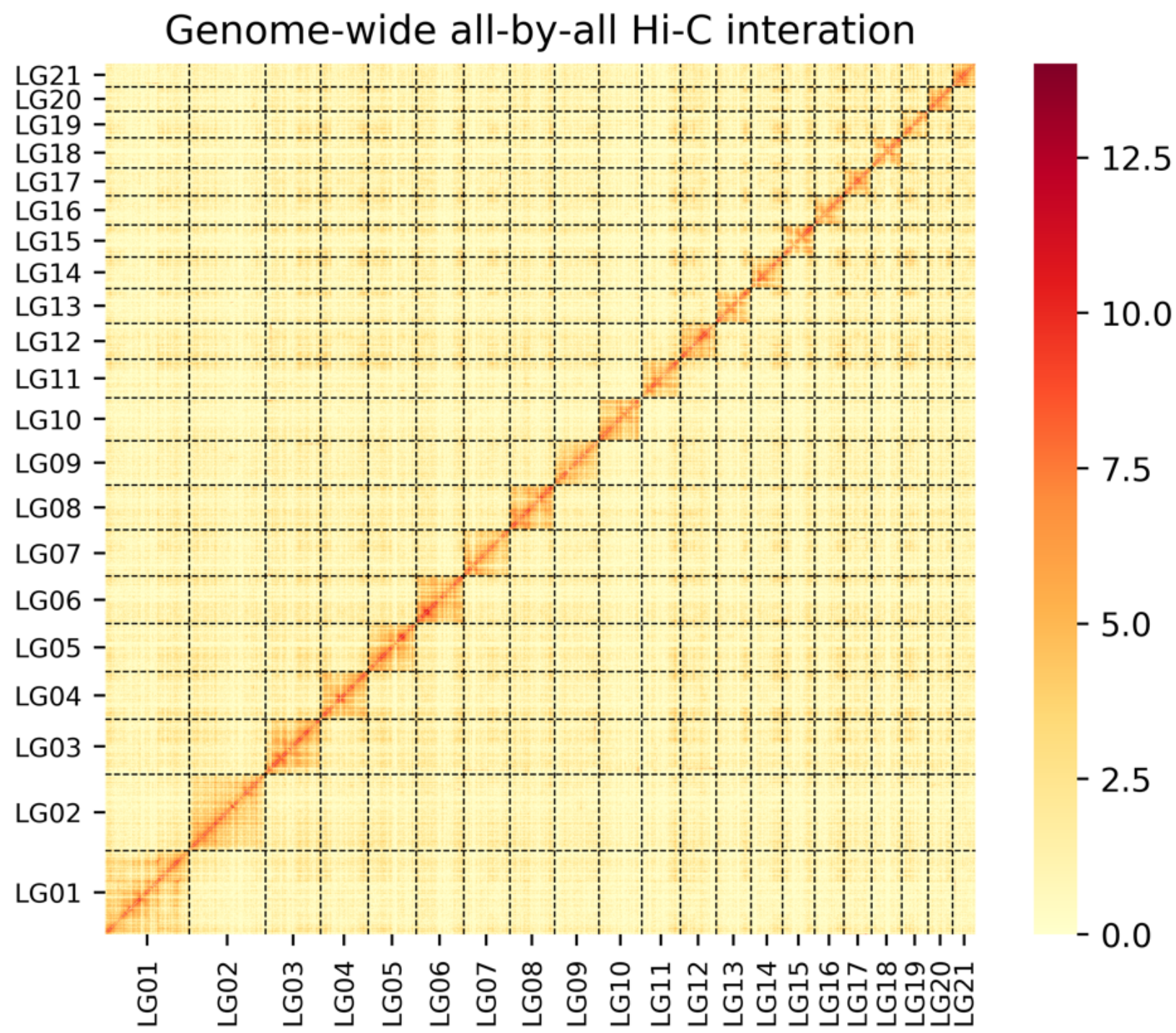

Supplementary Fig S1. Hi-C interaction map of 21 pseudo-chromosomes (chr) of *Acanthaster planci* genome performed by Juicebox. Strong interactions were indicated in dark red.

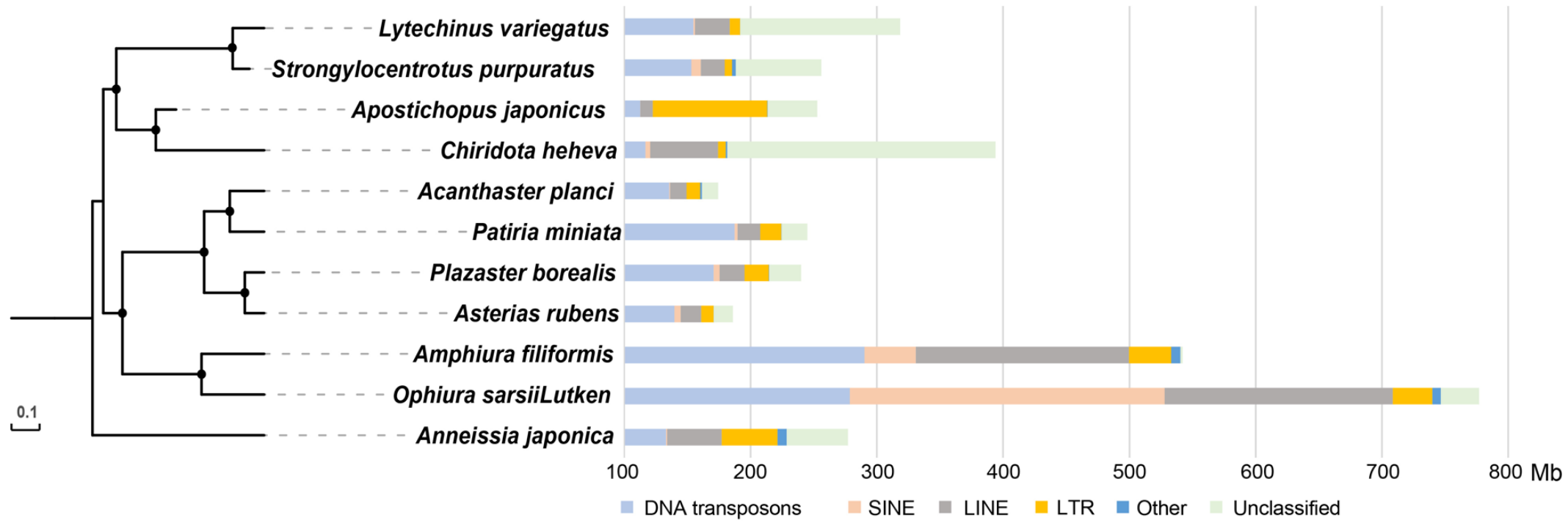

Supplementary Fig S2. Total length of TE elements in selected echinoderms. Black circles on nodes indicate 100% bootstrap support.

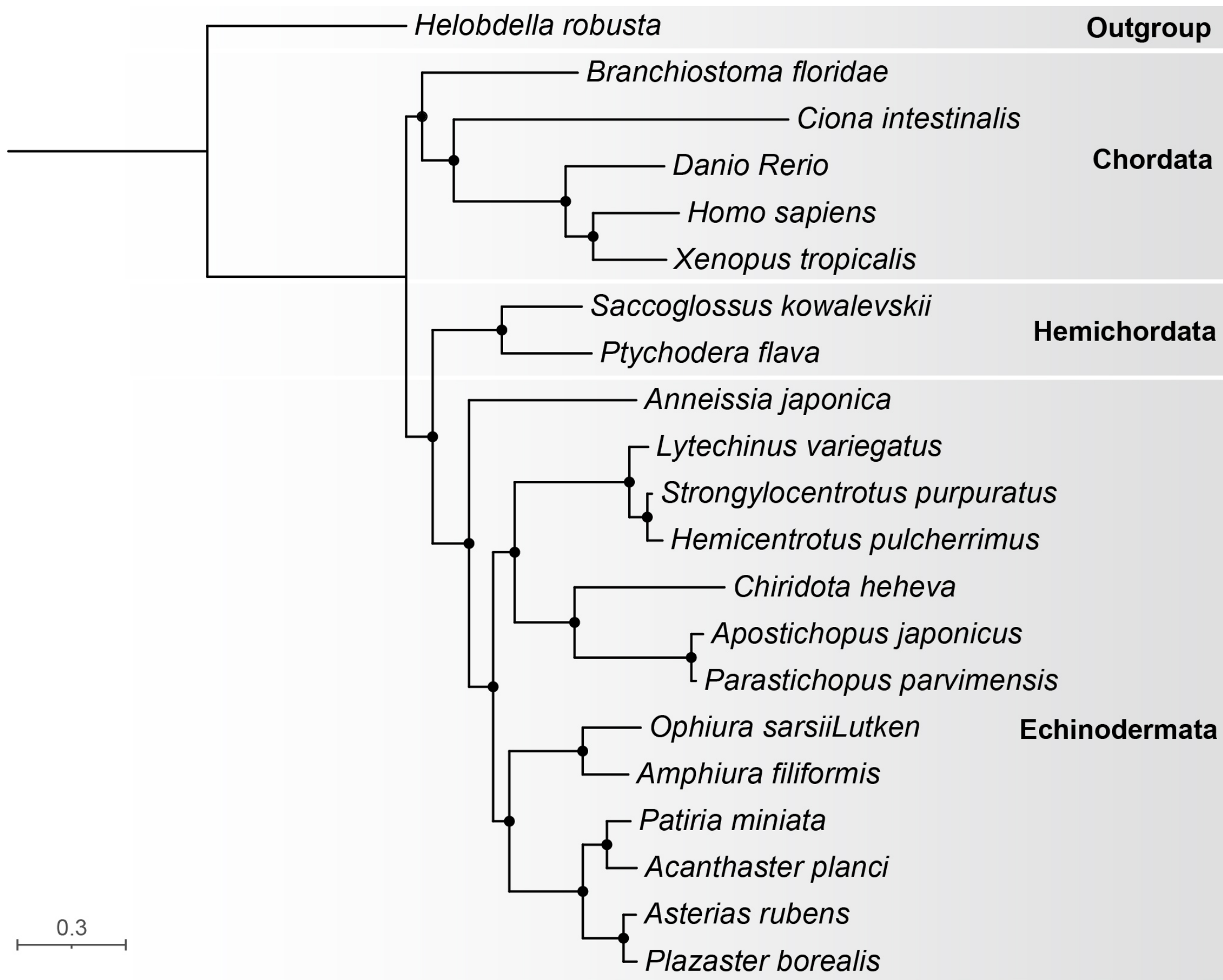

Supplementary Fig S3. Maximum-likelihood phylogenetic relationships among 18 selected metazoans. The tree is constructed LG+I+G model with 1,000 bootstrap replicates. Black circles on nodes indicate 100% bootstrap support.

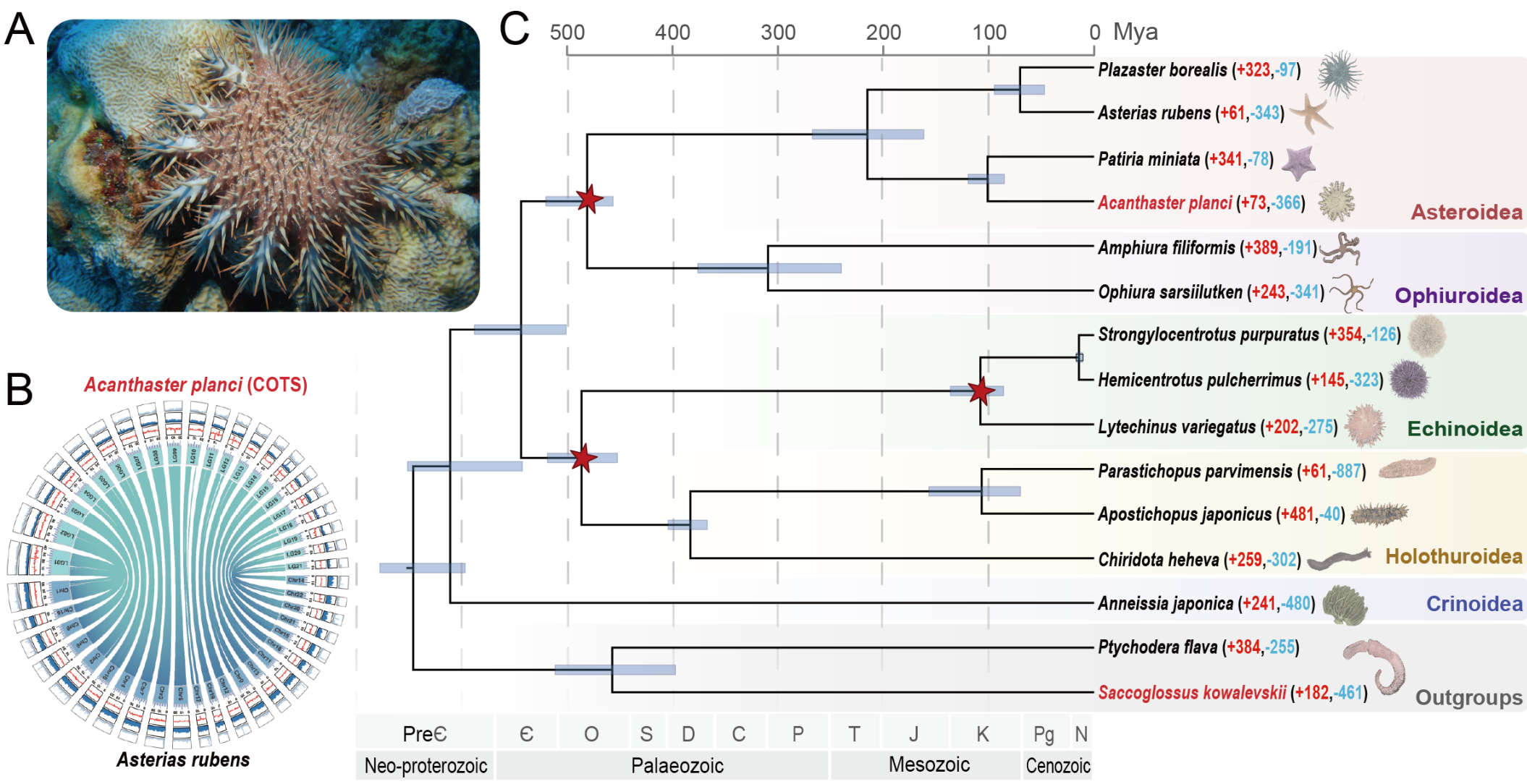

Supplementary Fig S4. (A) Adult COTS predating on coral. (B) Syntenic relationship and Genome overview of *Asterias rubens* and *Acanthaster planci.* Tracks from inner to outer represent GC skew, repeats content density, genes density, and assembled chromosomes, respectively, with densities calculated within a 500-kb window. (C) Phylogenetic tree showing the phylogenetic position of *A. planci* in deuterostome. The tree was constructed using a maximum likelihood method with LG+I+G model and calibrated with fossil records at four nodes shown with a red star. The purple lines on the nodes indicate divergence time with a 95% confidence interval. The numbers on each branch indicate gene family expansion (red) and contraction (green). C, Carboniferous; Є, Cambrian; D, Devonian; J, Jurassic; K, Cretaceous; M, Mesozoic; N, Neogene; O, Ordovician; P, Permian; Pg, Paleogene; PreЄ, Precambrian; S, Silurian; T, Triassic.

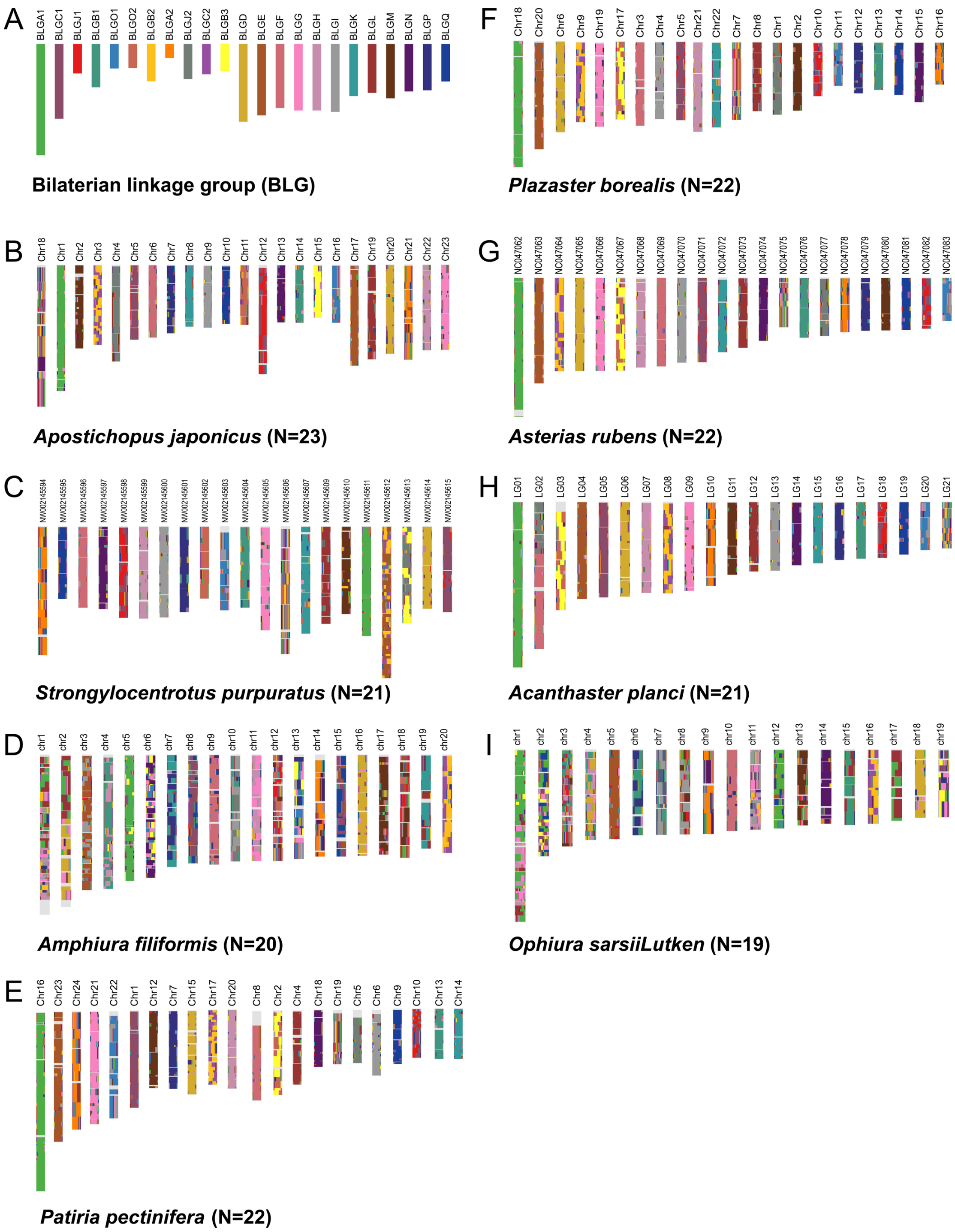

Supplementary Fig S5. Bilaterian linkage group (BLG) orthologs on echinoderm chromosomes. (A) BLG color assignments. (B-I) BLG orthologues on selected echinoderm chromosomes. Echinoderm chromosomes show some fusion of BLGs, but most of them retain their original structure. Some BLG fusion events might have happened in echinoderm ancestors: BLGC1 and BLGJ1, BLGI and BLGO1, BLGC2 and BLGQ.

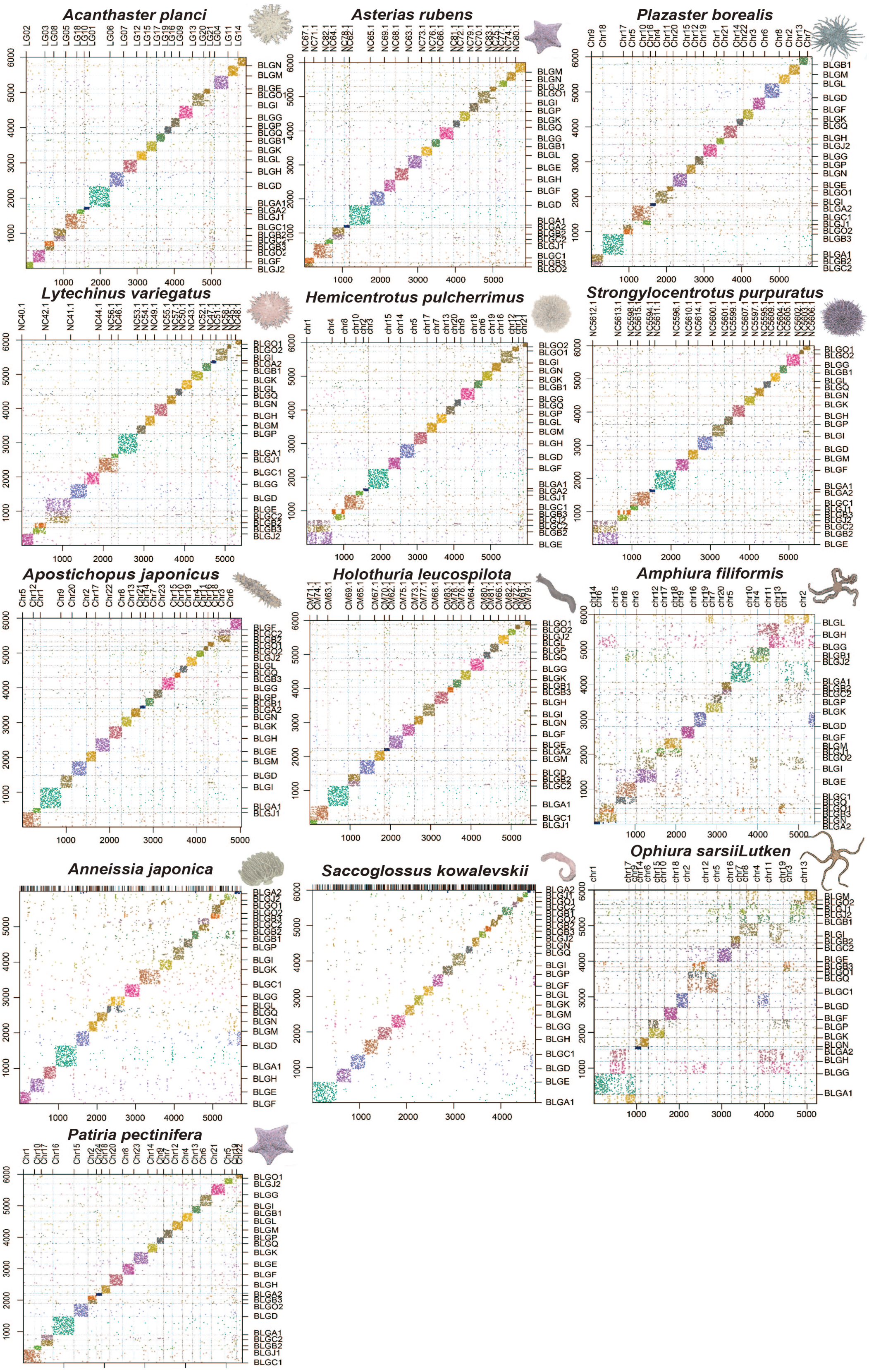

Supplementary Fig S6. Conservation and disruption of synteny in deuterostomes. Dot plots show the syntenies between deuterostome genome sequences and BLGs.

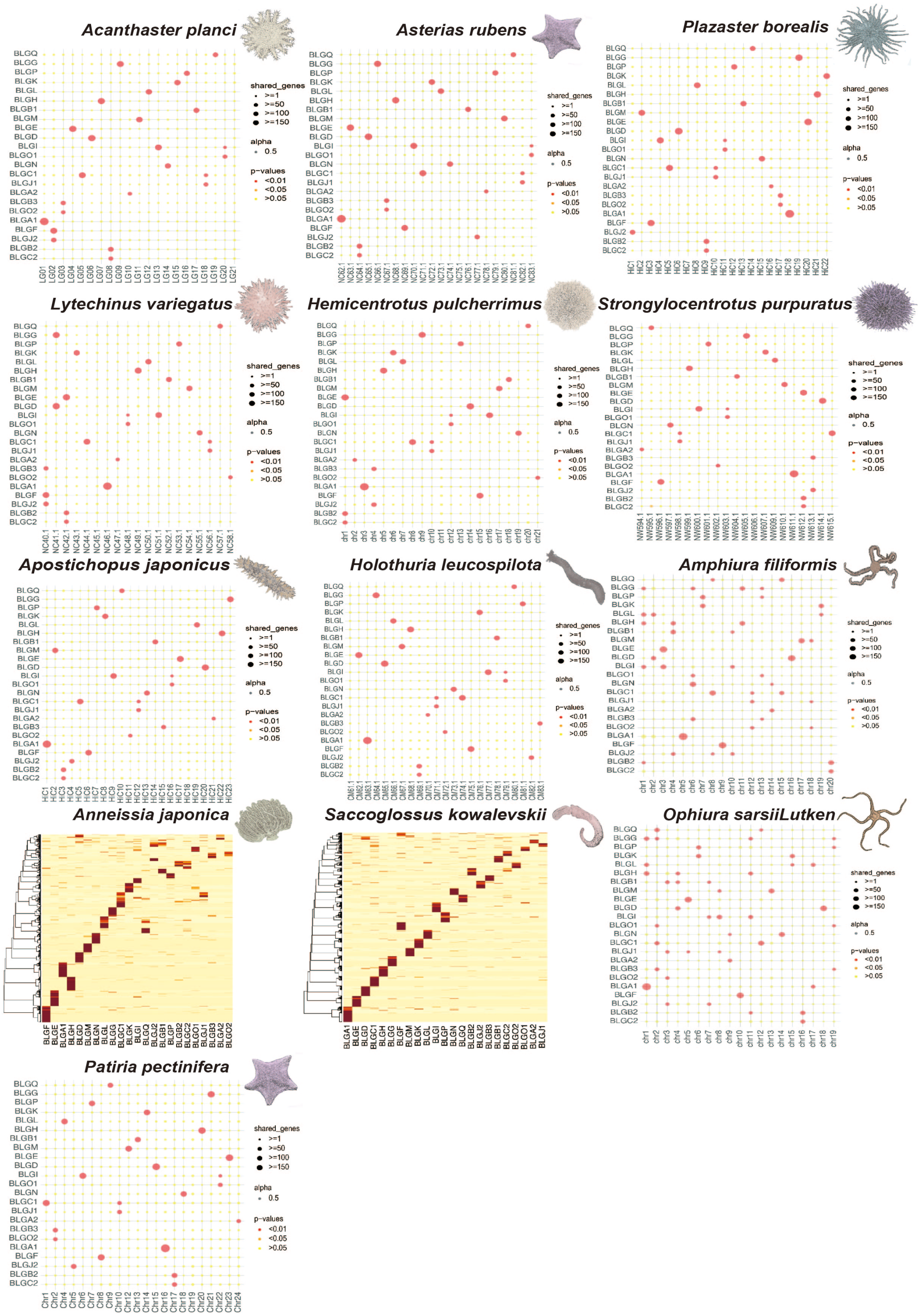

Supplementary Fig S7. Fisher plots (one-sided, alternative "greater", Bonferroni multiple test corrected) showing enrichment of orthologs for each BLG on chromosomes of six species. Size of dots corresponds to the number of shared orthologs between a given BLG and the chromosome of a species. P-values are color coded. For fragmented genome, heatmap (Light yellow background) was using to cluster scaffolds to reconstruct BLG structure in those species.

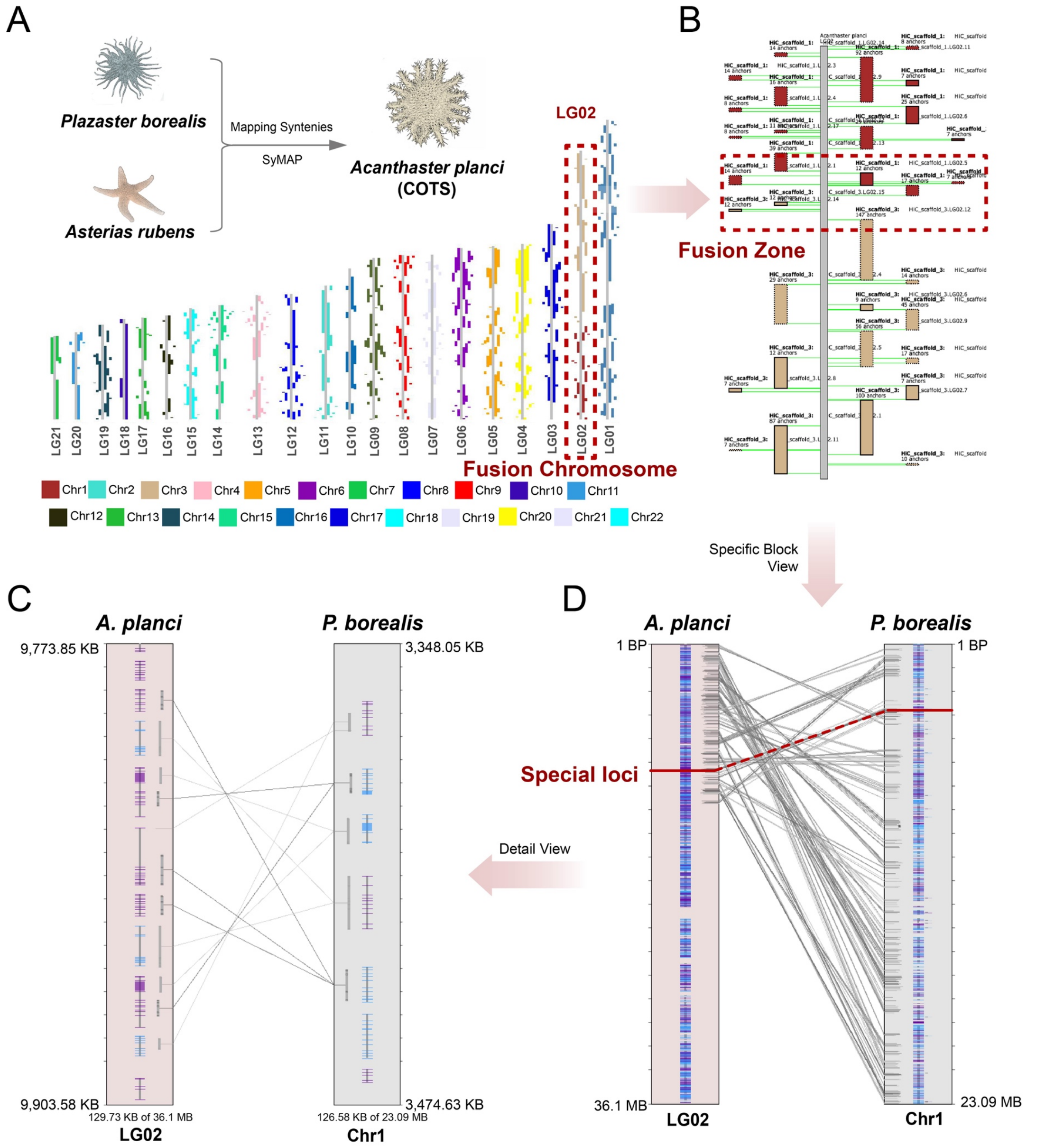

Supplementary Fig S8. The workflow of chromosome-scale macro-synteny comparision using SyMAP v3.4. (A) block view of syntenies between *Acanthaster planci* and *Plazaster borealis* genome. (B) Block detail view of *A. planci* pseudo-chromosomes LG02. (C) The special duplication region in pseudo-chromosomes LG02 (9.65-9.90 Mb). (D) Specific block view between *P. boreali*: pseudo-Chr1 and *A. planci*: LG02. The same workflow also used between *A. planci and A. rubens.*

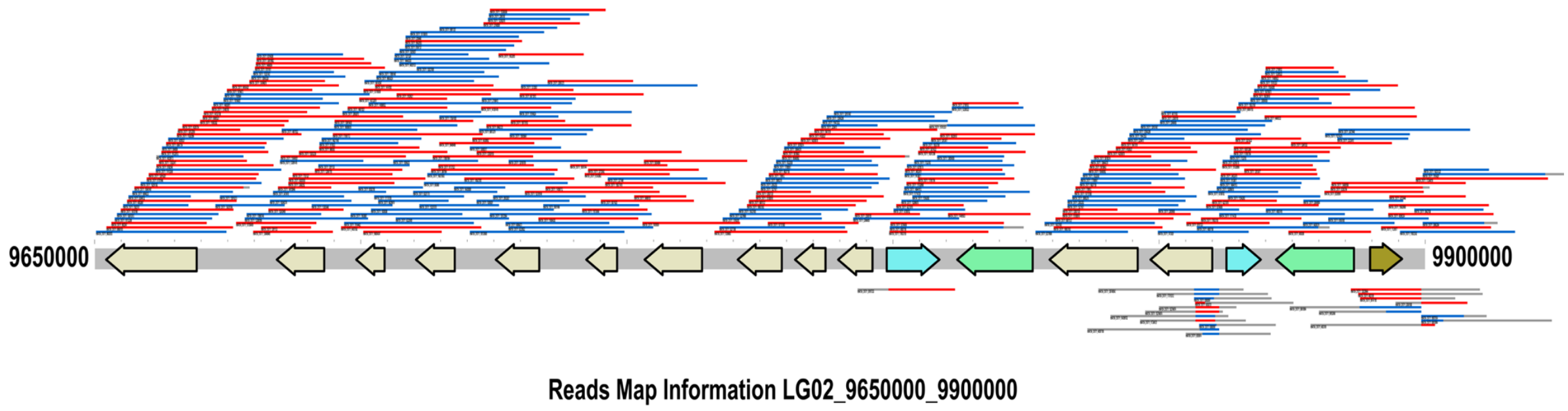

Supplementary Fig S9. HiFi reads map information in LG02 special gene duplication region. Reads sequenced from sense strand are labelled in red and reads sequenced from antisense strand are labelled in blue.

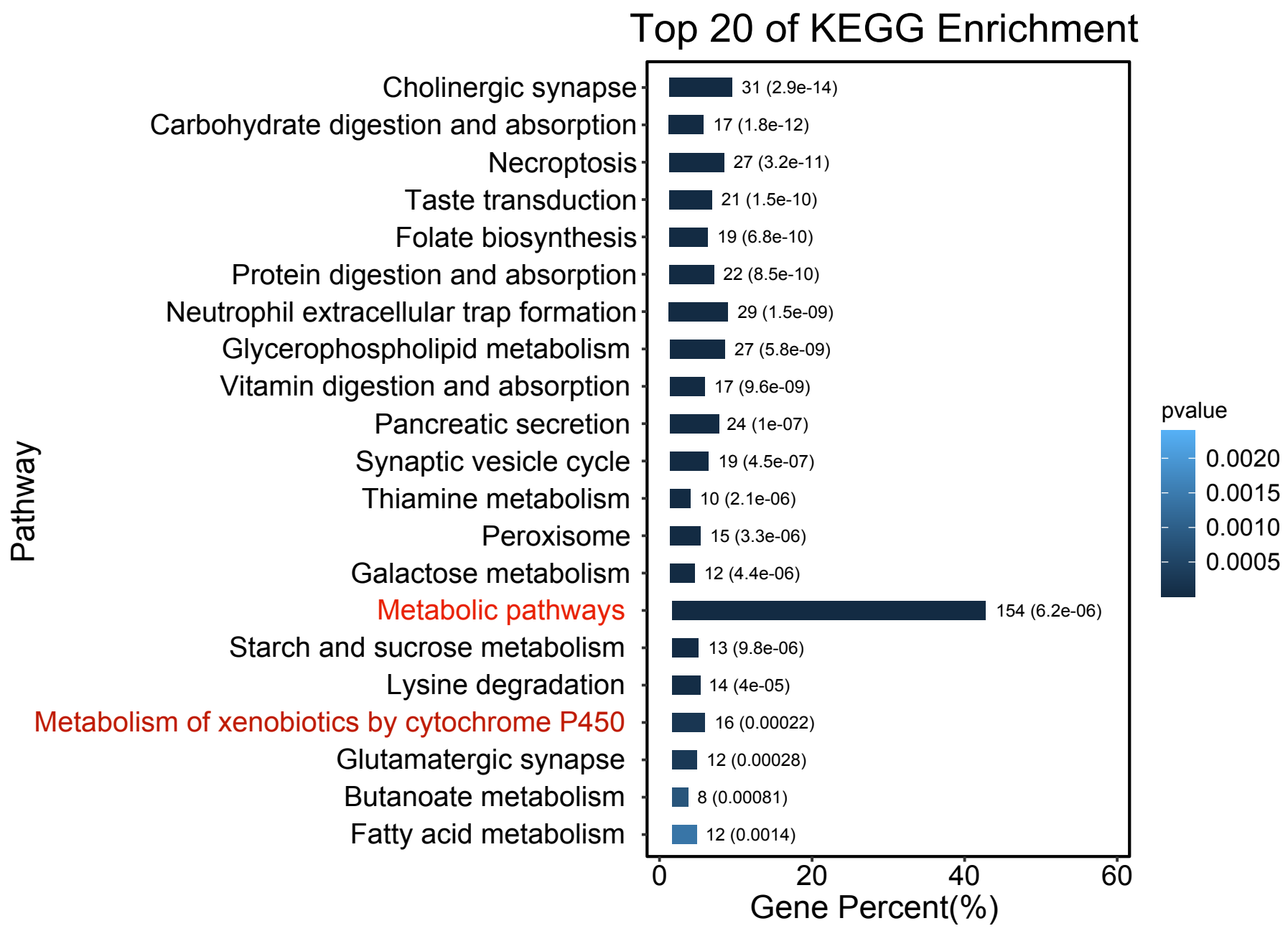

Supplementary Fig S10. KEGG enrichment result of expanded gene families in *Acanthaster planci* genome.

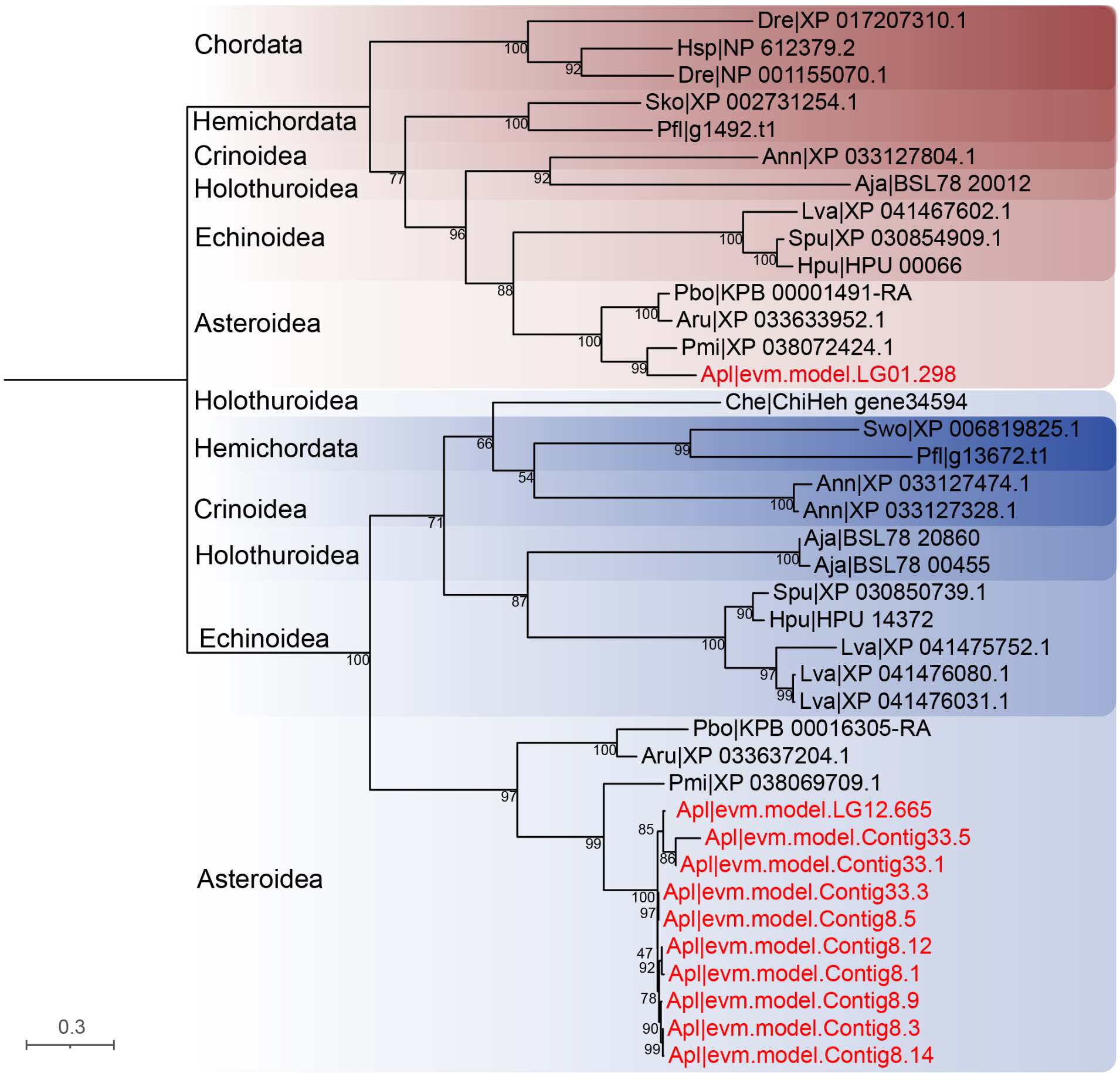

Supplementary Fig S11. Phylogenetic tree of PKDCC in selected metazoans. Sequences of *Acanthaster planci* are labelled in red.

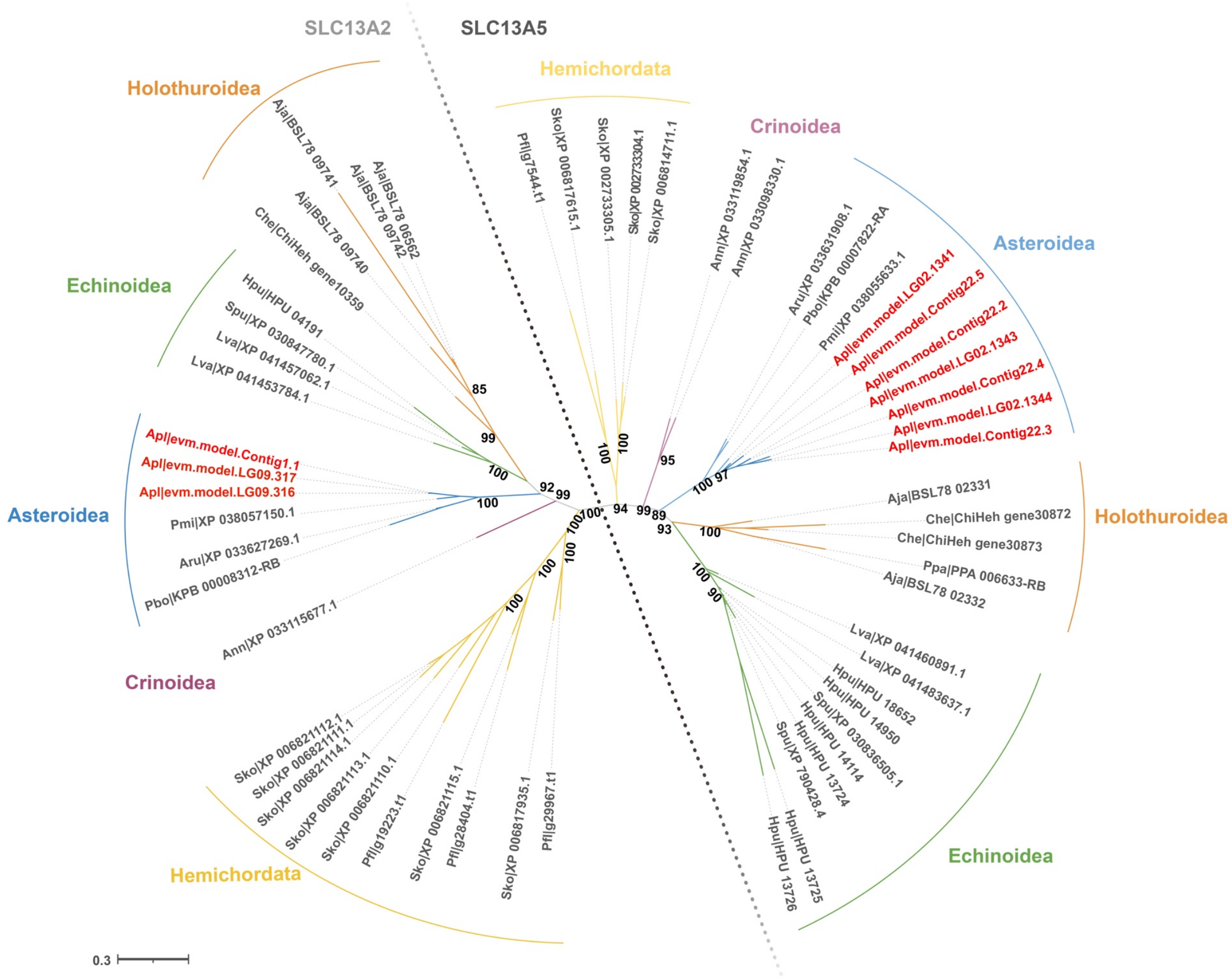

Supplementary Fig S12. Phylogenetic tree of SLC13A5/A2 in selected metazoans. Sequences of *Acanthaster planci* are labelled in red.

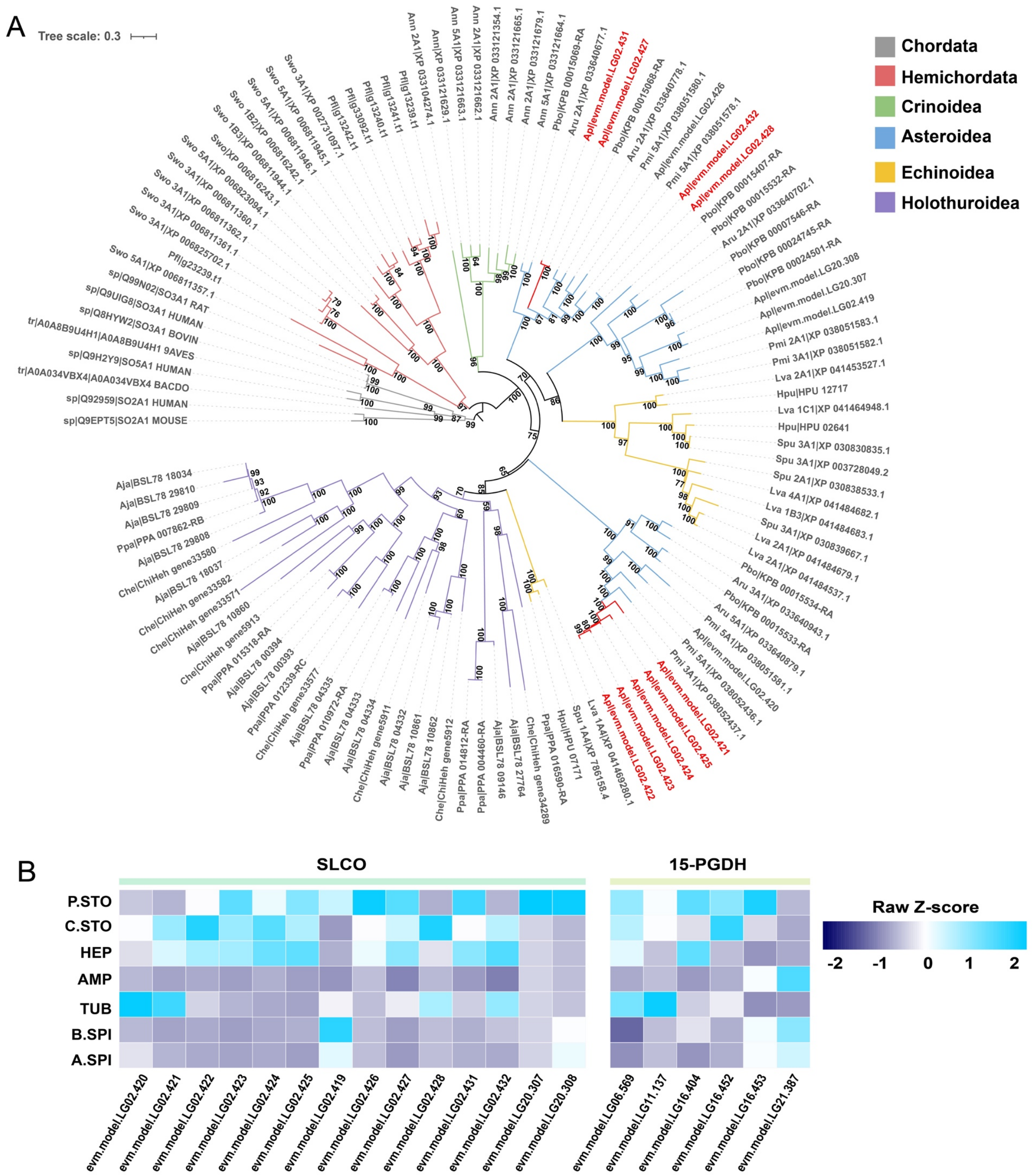

Supplementary Fig S13. Expansion and expression of SLCO genes in *Acanthaster planci.* (A) Phylogenetic tree of SLCO in selected metazoans. Expanded SLCO genes of *A. planci* are labelled in red. (B) Expression profile of SLCO and 15-PGDH in seven tissues.

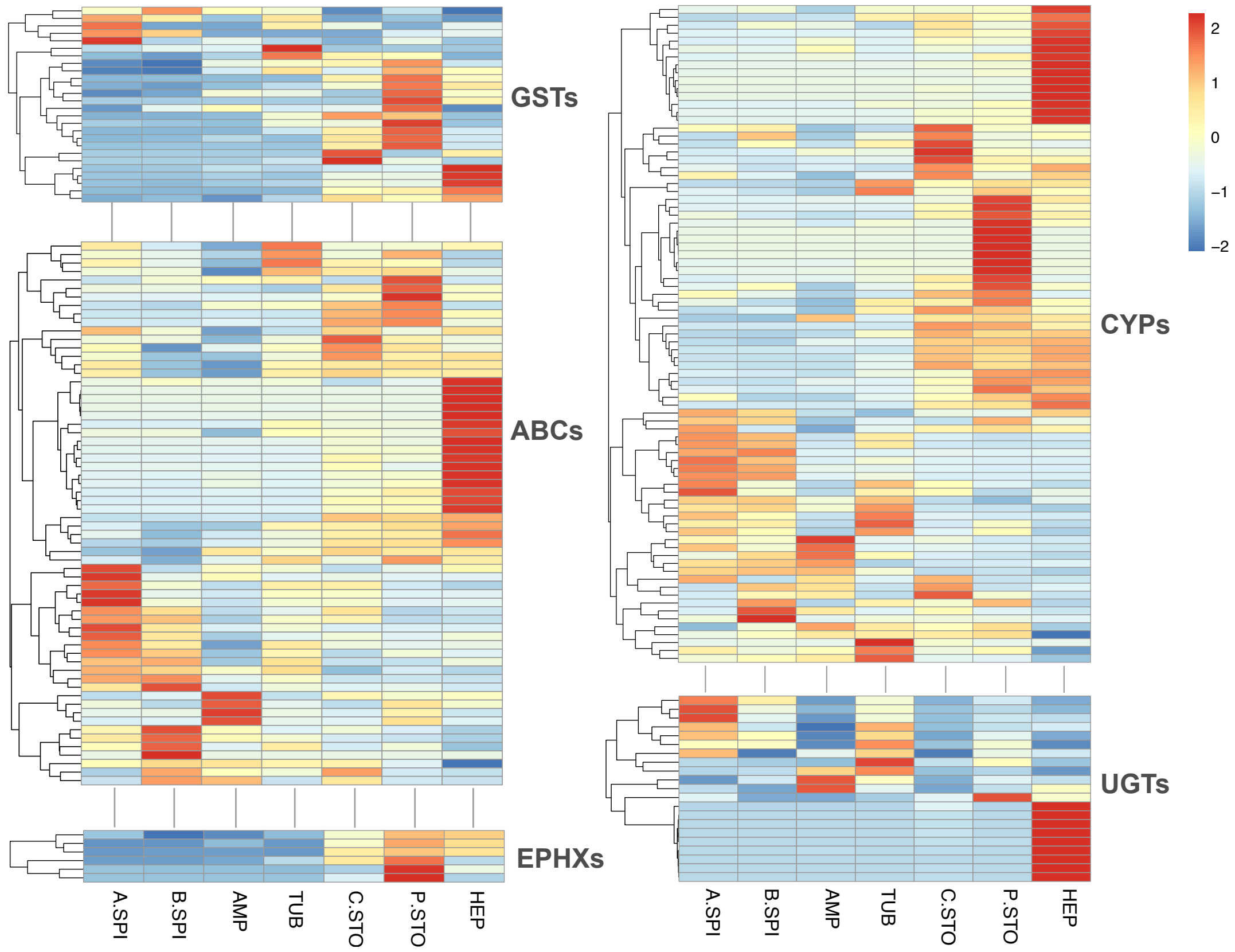

Supplementary Fig S14. Expression patterns of detoxification-related genes across tissues. HEP: Hepatic cecum; A.SPI: Spine apex; B.SPI: Spine base; AMP: Ampullae; C.STO: Central stomach; P.STO: pyloric stomach; TUB: Tubefeet.

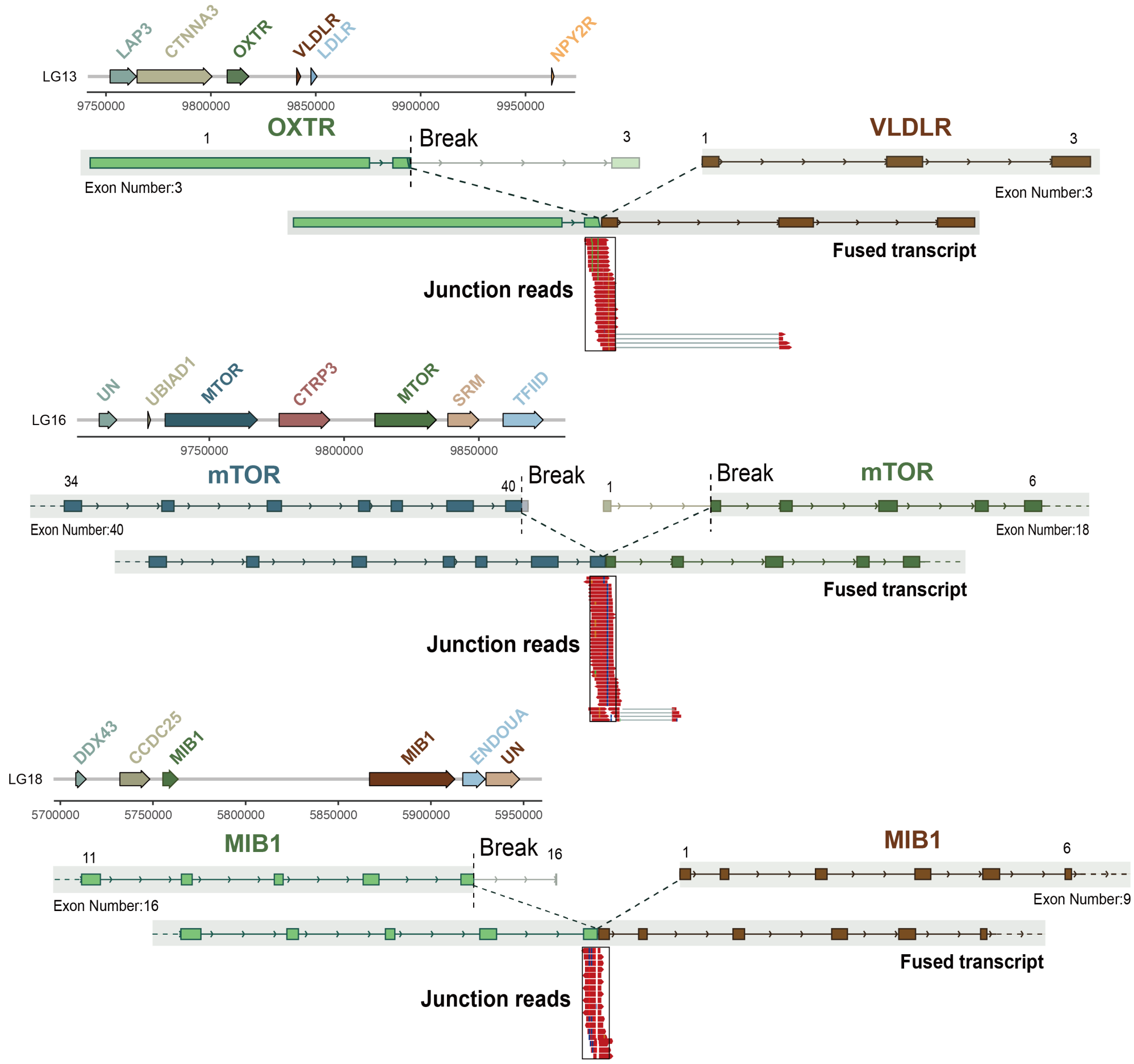

Supplementary Fig S15. Stacks of sequencing reads from Integrated Genome Viewer spanning the breakpoints of the fusion transcripts. The dotted line in the middle of the diagram represents the location of the breakpoint. As there are too many exons, Figures of mTOR and MIB1 fusion transcripts only show exon structures near the breakpoint.

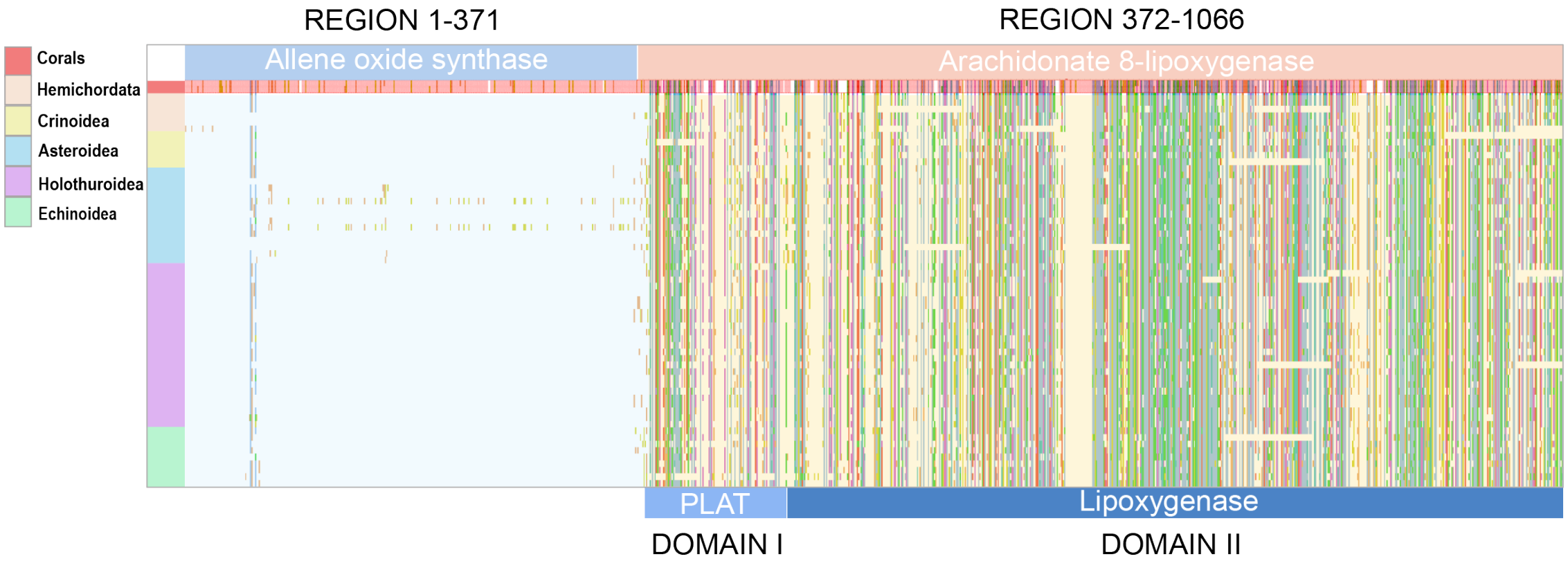

Supplementary Fig S16. Protein alignment of AOSL in corals and echinoderm. The red line at the top represents the complete sequence structure of coral AOSL with two regions, Allene oxide synthase and Arachidonate 8-lipoxygenase.

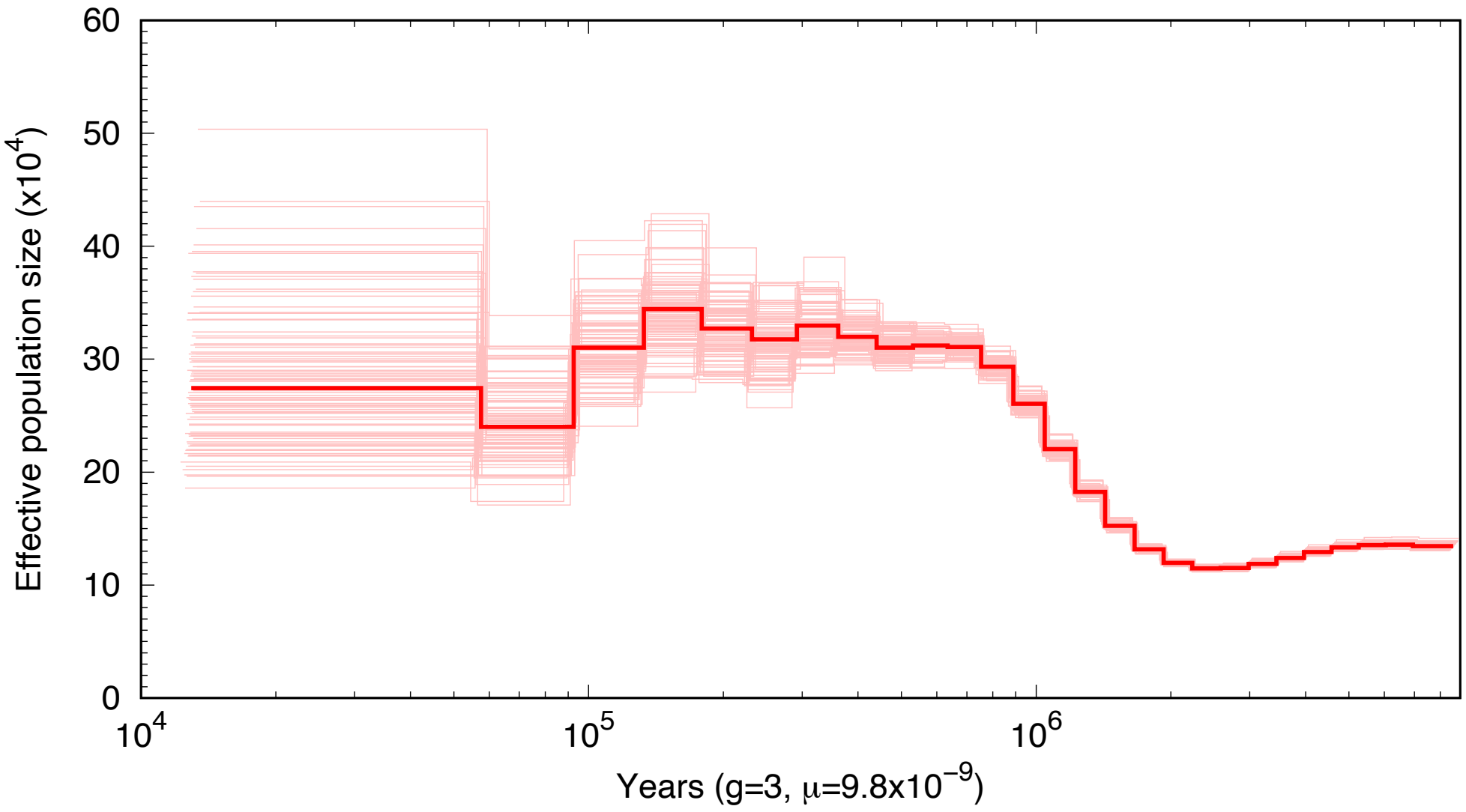

Supplementary Fig S17. Historical effective population sizes inferred from COTS genomes in the South China Sea, assuming a generation time of 3 years and a substitution mutation rate of 9.8e-9 per generation.

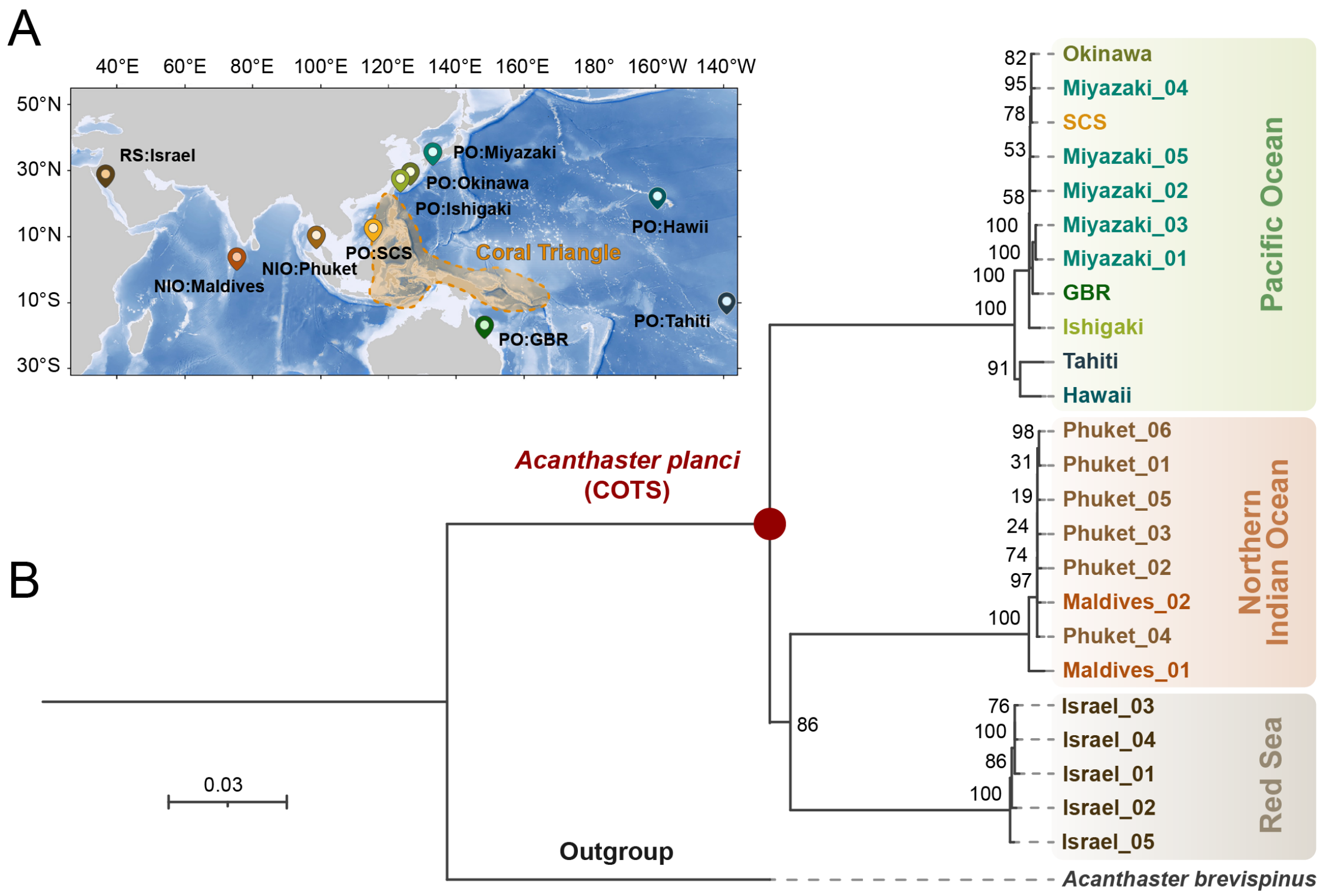

Supplementary Fig S18. Maximum likelihood (ML) phylogeny in *Acanthaster planci*. NIO, RS, and PO represent COTS species (NIO: Northern Indian Ocean species, RS: Red Sea species, and PO: Pacific Ocean species). (A) The location of *A. planci* from three regions: are labelled in the map. (B) ML tree based on 14 conserved coding gene of the mitochondrial genome of 24 COTS and '*Acanthaster brevispinus*' as outgroup species.

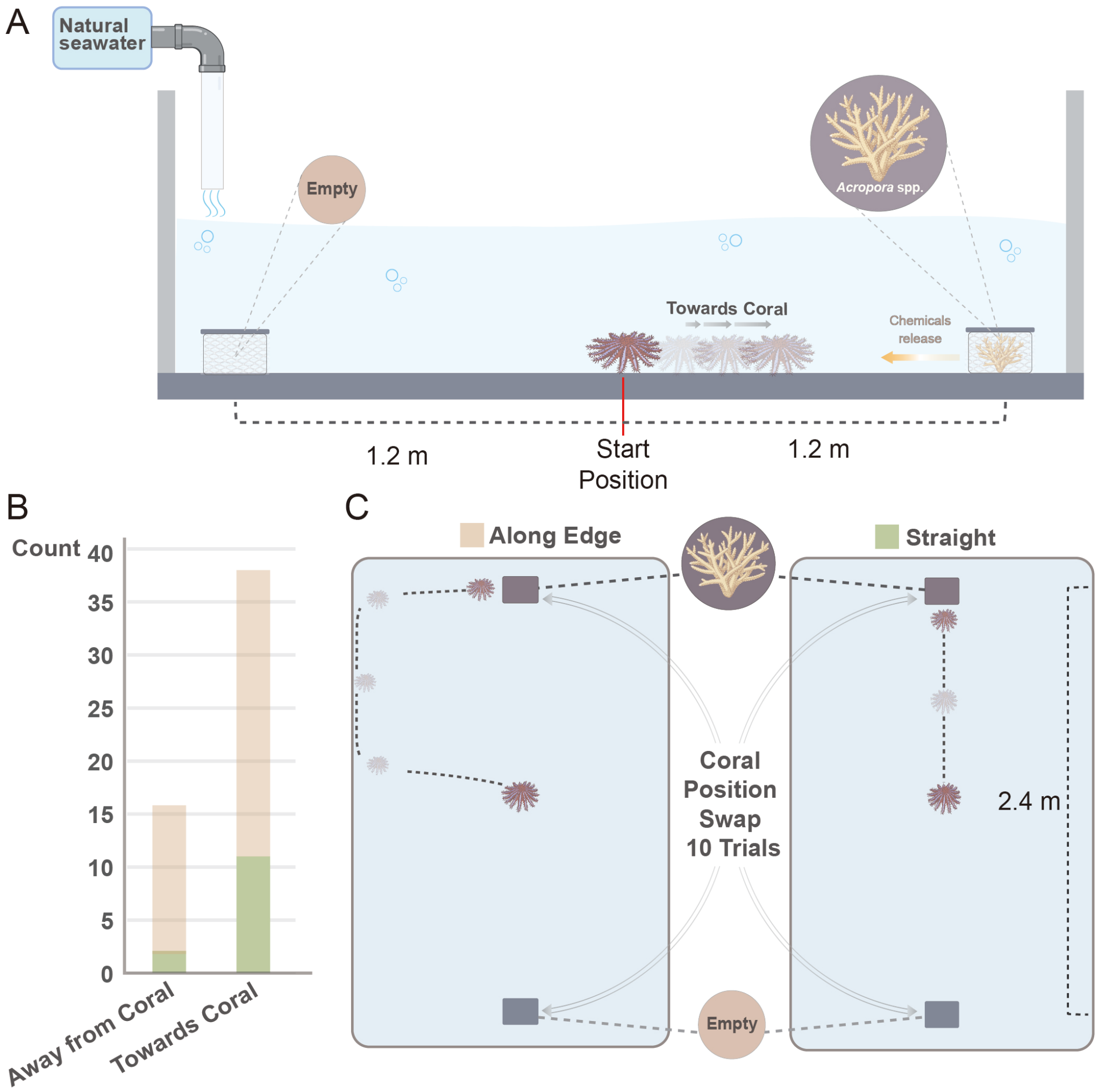

Supplementary Fig S19. Schematic of behavioral induction experiment in crown-of-thorns starfish. (A) Schematic of the experimental setup. In each trial, the direction and trajectory of crown-of-thorns starfish movement from the starting point over a set time (15 minutes) were recorded. Movements were classified as 'Away from Coral' or 'Towards Coral.' (B) Statistical count of crown-of-thorns starfish crawling directions. The number of movements towards the coral was higher than those away from it, and the number of starfish moving in a straight line towards the coral was significantly higher ($p < 0.05$, Total number of trials: 60). (C)Two movement patterns of crown-of-thorns starfish. The left panel shows the starfish moving towards the coral along the tank edge, while the right panel shows the starfish moving directly towards the coral.

**Sequencing Platform**	**Library Length**	**Read Length**	**Raw reads/READ**	**Raw bases**	**Accession Number**
Illumina HiSeq	350	2x 150	228,924,674	34,455,468,000	PRJNA1039947
Pacbio Sequel II	Non fragmented genomic DNA	>2000	582,242	11,606,764,784	PRJNA1039947
Hi-C BGI MGISEQ-2000	Hi-C	2x 150	709,023,720	106,020,798,972	PRJNA1039947

Supplementary Table S1. Sequencs for assembly of the *Acanthaster planci* genome.

Assembly Feature	Statistics
Number of scaffolds	288
Total assembly size (bp)	437,159,099
21 pseudo-chromosomes + 7375 contigs	417,149,432 + 20009667
Longest scaffolds (nt)	40,176,291
N50 scaffold length (nt)	21,642,976
L50 scaffold count	9
N50 contig length (nt)	1,376,456
L50 contig count	98
Length of each pseudo-chromosome (bp)	
LG01	40,170,491
LG02	36,092,812
LG03	26,171,859
LG04	23,533,906
LG05	23,250,855
LG06	22,969,945
LG07	22,145,678
LG08	22,093,390
LG09	21,640,476
LG10	20,276,873
LG11	18,011,014
LG12	16,849,622
LG13	16,700,598
LG14	15,368,971
LG15	14,931,518
LG16	13,975,374
LG17	13,714,196
LG18	13,520,610
LG19	12,752,393
LG20	11,748,218
LG21	11,230,633
BUSCO assessment results	C:97.7%[D:6.3%], F:1.6%, M:0.7%

Supplementary Table S2. Summary statistics of the genome assembly and annotation.

		Phuket01	Israel04	Miyazaki01	This Study
**Assembly size (bp)**		400438766	385630944	420016408	437219299
	Scaffold number	3642	3552	4526	288
	Scaffold N50 (bp)	3897460	1662177	2941429	21642976
	Max Scaffold length (bp)	11313316	8528531	10098247	40176291
	Total number of Ns (bp)	7197764	7359702	4264832	60202
	GC content (%)	41.4	41.4	41.3	41.48
	Heterozygosity (%)	0.72	1.24	0.97	0.49
**Genome evaluation (BUSCO)**	Complete (%)	98.0	97.7	98.1	97.1
	Complete and single-copy (%)	97.6	97.4	97.6	93.5
	Complete and duplicated (%)	0.4	0.3	0.5	3.6
	Fragmented (%)	0.7	0.9	1.4	1.7
	Missing (%)	1.3	1.4	0.5	1.2
**Predicted Proteins**	Gene number	18045	18879	19397	16918
**Protein evaluation (BUSCO)**	Complete (%)	98.2	98.0	98.1	97.7
	Complete and single-copy (%)	97.8	97.8	97.6	91.4
	Complete and duplicated (%)	0.4	0.2	0.5	6.3
	Fragmented (%)	1.3	1.3	1.3	1.6
	Missing (%)	0.5	0.7	0.6	0.7
**Platform**		Illumina	Illumina	Illumina	HI-C
**CEGMA**					
	Total number of core genes queried	248	248	/	248
	Complete	180(72.58%)	182(73.39%)	/	178(71.77%)
	Partial	239(96.37%)	235(94.76%)	/	234(94.35%)
	Average number of orthologs per core genes	1.42	1.41	/	1.53
	Number of missing core genes	9(3.62%)	13(5.24%)	/	14(5.64%)
	% of detected core genes that have more	34.73	34.89	/	40.6

than 1 ortholog

Supplementary Table S3. Assessment of the COTS genomes (three published genome and one in this study).

	Number	Length（bp）	Percentage（%）
**Retroelements**	174331	49184590	11.25
**SINEs:**	12028	1597323	0.37
Penelope	5200	1263677	0.29
**LINEs:**	78028	25893234	5.92
CRE/SLACS	0	0	0
L2/CR1/Rex	9387	3062490	0.7
R1/LOA/Jockey	231	324558	0.07
R2/R4/NeSL	91	41557	0.01
RTE/Bov-B	1544	593402	0.14
L1/CIN4	981	64215	0.01
**LTR:**	84275	21694033	4.96
BEL/Pao	1210	827376	0.19
Ty1/Copia	0	0	0
Gypsy/DIRS1	7993	5203738	1.19
Retroviral	0	0	0
**DNA transposons ：**	326470	71061757	16.25
hobo-Activator	1301	686944	0.16
Tc1-IS630-Pogo	1423	418273	0.1
PiggyBac	0	0	0
Tourist/Harbinger	307	63739	0.01
Other(Mirage,P-element,Transib)	382	213818	0.05
**Rolling circles**	0	0	0
**Unclassified:**	127531	25773484	5.89
**Small RNA:**	13	2166	0
**Satellites:**	0	0	0
**Simple repeats:**	42201	2886450	0.66
**Low complexity**	6824	355423	0.08

Supplementary Table S4. Repetitive elements in the *Acanthaster planci* genome.

Type		Number	Percent (%)
Swissprot		14,003	82.77
KEGG		10,113	59.78
KOG		11,339	67.02
GO		10,551	62.37
BLASTNR		14,757	87.23
PANTHER		14676	84.34
Total	Annotated	15,246	86.74
	Gene	16,918	-

Supplementary Table S5. Summary of functional annotation of the *Acanthaster planci* genome.

Taxon	Three letter code	species	ID	Reference
Enteropneusta	Pfl	*Ptychodera flava*	Pfl ver3.0	[36]
Enteropneusta	Sko	*Saccoglossus kowalevskii*	GCA_000003605.1	[36]
Crinoidea	Ann	*Anneissia japonica*	GCA_011630105.1	[37]
Echinoidea	Lva	*Lytechinus variegatus*	GCA_011630205.1	[37]
Echinoidea	Hpu	*Hemicentrotus pulcherrimus*	GCA_003118195.1	[38]
Echinoidea	Spu	*Strongylocentrotus purpuratus*	GCA_000002235.4	[39]
Asteroidea	Aru	*Asterias rubens*	GCA_902459465.3	PRJNA68306
Asteroidea	Pbo	*Plazaster borealis*	GCA_021014325.1	[40]
Asteroidea	Pmi	*Patiria miniata*	GCA_015706575.1	[41]
Asteroidea	Ppe	*Patiria pectinifera*	JASAOE000000000	[42]
Holothuroidea	Aja	*Apostichopus japonicus*	GCA_002754855.1	[43]
Holothuroidea	Ppa	*Parastichopus parvimensis*	GCA_000934455.1	[41]
Holothuroidea	Che	*Chiridota heheva*	GCA_020152595.1	[44]
Holothuroidea	Hle	*Holothuria leucospilota*	GCA_029531755.1	[45]
Ophiuroidea	Afi	*Amphiura filiformis*	/	[46]
Ophiuroidea	Osa	*Ophiura_sarsii Lutken*	GCA_035610835.1	[47]
Chordata	Dre	*Danio rerio*	GCF_000002035.6	[48]
Chordata	Hsp	*Homo sapiens*	GCF_000001405.40	[49]
Chordata	Xtr	*Xenopus tropicalis*	GCF_000004195.4	[50]
Chordata	Cin	*Ciona intestinalis*	GCF_000224145.3	[51]
Chordata	Bfl	*Branchiostoma floridae*	GCA_000003815.2	[52]
Annelida	Hro	*Helobdella robusta*	GCF_000326865.2	[53]

Supplementary Table S6. Genome assemblies used in comparative analyses.

FamilyID	Change index	Function	p-value
OG0000027	+2	metabotropic glutamate receptor-like	0
OG0000030	+3	cholinesterase 1	0
OG0000036	+9	histone H2A	0
OG0000049	+4	carboxypeptidase B	0
OG0000055	+3	cytochrome P450 4F-like	0
OG0000063	+2	sodium-coupled monocarboxylate transporter 1-like	0
OG0000107	+2	dynein heavy chain	0.006
OG0000170	+2	glutathione S-transferase theta-1-like	0
OG0000191	+5	very long-chain acyl-CoA synthetase-like	0.002
OG0000198	+2	alpha-amylase	0
OG0000206	+2	ileal sodium/bile acid cotransporter-like	0
OG0000234	+4	carbonyl reductase [NADPH] 1-like	0
OG0000260	+6	solute carrier family 13 member 5/2 -like	0
OG0000271	+3	multidrug resistance-associated protein 4-like	0
OG0000278	+3	solute carrier organic anion transporter family member 5A1	0
OG0000285	+2	unclear	0
OG0000296	+2	protein dispatched homolog 1	0.016
OG0000356	+3	putative methyltransferase DDB_G0268948	0
OG0000563	+2	unclear	0.02
OG0000643	+2	arylsulfatase	0.001
OG0000671	+2	component C3	0.001
OG0000682	+2	peptidoglycan-recognition protein SC2-like	0.018
OG0000697	+2	collagen alpha-1(I) chain-like	0
OG0000998	+3	choline dehydrogenase, mitochondrial	0.018
OG0001104	+2	pancreatic triacylglycerol lipase-like	0.011
OG0001328	+8	extracellular tyrosine-protein kinase PKDCC-like	0
OG0001341	+2	unclear	0.001
OG0001526	+2	plancitoxin-1	0
OG0001757	+3	janus kinase and microtubule-interacting protein 2	0.035
OG0001760	+4	von Willebrand factor A domain-containing protein 2-like	0.029
OG0002244	+2	ketimine reductase mu-crystallin-like	0.003
OG0002958	+4	ras-related protein Rab-24-like	0.014
OG0003237	+3	teneurin-3	0.034
OG0004359	+5	tubulin alpha chain-like	0
OG0004679	+2	annexin A6-like	0.006
OG0006208	+2	snurportin-1-like	0.008

OG0006680	+3	guanine nucleotide-binding protein G(I)/G(S)/G(O) subunit gamma-10	0.001
OG0009189	+7	zinc finger protein 77-like	0.014
OG0010259	+7	unclear	0.007
OG0010801	+2	proton-coupled folate transporter-like	0.033

Supplementary Table S7. Expanded gene families in *A. planci* genome.

Gene id	Symbol	Selection Site	Site AA
Apl|evm.model.LG15.513	MAMDC2	84	I
Apl|evm.model.LG02.385	HAO1	190	F
Apl|evm.model.LG09.91	MSI2	297	Y
Apl|evm.model.LG03.664	GAL3ST1	608	G
Apl|evm.model.LG09.116	-	3925	R
Apl|evm.model.LG21.280	-	43	G
Apl|evm.model.LG15.128	-	2311	S
Apl|evm.model.LG08.9	COL2A1	605	P
Apl|evm.model.LG05.673	FK506-bp1	189	S
Apl|evm.model.LG18.336	-	583	W
Apl|evm.model.LG13.241	DDX58	363	C
Apl|evm.model.LG08.72	DNASE1	23	T
Apl|evm.model.LG10.425	-	2689	C
Apl|evm.model.LG04.502	ACOX1	253	S
Apl|evm.model.LG07.26	Pef1	163	K
Apl|evm.model.LG10.120	-	69	K
Apl|evm.model.LG14.739	CASK	376	F
Apl|evm.model.LG15.366	REV3L	750	R
Apl|evm.model.LG11.614	-	256	S
Apl|evm.model.LG18.83	RTEL1	614	R
Apl|evm.model.LG20.15	-	55	R
Apl|evm.model.LG05.471	-	194	N
Apl|evm.model.LG21.179	KIAA1919	225	K
Apl|evm.model.LG02.1422	-	1	E
Apl|evm.model.LG13.640	HYAL1	60	L
Apl|evm.model.LG03.166	KCP	146	V
Apl|evm.model.LG03.548	FZD7	171	V
Apl|evm.model.LG14.266	-	137	S
Apl|evm.model.LG01.1449	-	33	R
Apl|evm.model.LG15.297	ENPP4	317	L
Apl|evm.model.LG19.59	PRKG1	12	G
Apl|evm.model.LG01.960	DLL1	19	W
Apl|evm.model.LG07.36	DNAH9	963	Q
Apl|evm.model.LG02.169	SOGA3	335	A
Apl|evm.model.LG13.3	-	17	M
Apl|evm.model.LG01.420	-	172	S
Apl|evm.model.LG12.606	KIF2A	4	Q
Apl|evm.model.LG19.505	HGSNAT	412	E
Apl|evm.model.LG02.1091	PCM1	1112	D
Apl|evm.model.LG02.1235	RAD50	420	G
Apl|evm.model.LG10.559	SORL1	435	T

Apl|evm.model.LG11.208	-	69	A
Apl|evm.model.LG01.917	HNRNPU	690	A
Apl|evm.model.LG06.725	-	1774	S
Apl|evm.model.LG10.151	-	238	L
Apl|evm.model.LG10.227	ERG	484	N
Apl|evm.model.LG16.608	-	1318	S
Apl|evm.model.LG04.631	TTLL3	532	L
Apl|evm.model.LG07.747	CEP131	326	Q
Apl|evm.model.LG13.393	-	1131	W
Apl|evm.model.LG21.389	-	13	N
Apl|evm.model.LG01.547	SLC4A1AP	5	S
Apl|evm.model.LG02.1417	SQSTM1	5	Y
Apl|evm.model.LG03.650	DGKZ	6	E
Apl|evm.model.LG01.252	CEP128	4	Q
Apl|evm.model.LG01.958	INO80	902	S
Apl|evm.model.LG02.1039	BRCA2	813	S
Apl|evm.model.LG02.1412	SLC36A1	413	T
Apl|evm.model.LG02.630	NIPAL2	52	L
Apl|evm.model.LG07.903	PGAP1	1	C
Apl|evm.model.LG12.245	GTF3C3	56	S
Apl|evm.model.LG13.332	-	213	C
Apl|evm.model.LG19.3	GBF1	8	R
Apl|evm.model.LG20.181	CASC1	139	L
Apl|evm.model.LG01.1026	-	381	L
Apl|evm.model.LG01.455	XDH	186	M
Apl|evm.model.LG02.1404	VWA3B	2	E
Apl|evm.model.LG02.500	EEF1A1	119	S
Apl|evm.model.LG03.474	-	76	S
Apl|evm.model.LG05.331	ASH1L	2193	T
Apl|evm.model.LG07.369	-	329	S
Apl|evm.model.LG12.182	SPTLC1	339	L
Apl|evm.model.LG16.238	FTSJ1	280	E
Apl|evm.model.LG20.351	CAPRIN1	22	Q
Apl|evm.model.LG02.1125	-	283	F
Apl|evm.model.LG06.712	TRAPPC8	7	V
Apl|evm.model.LG07.166	rps9	2	F
Apl|evm.model.LG07.287	LENG8	330	S
Apl|evm.model.LG08.342	-	84	T
Apl|evm.model.LG08.411	-	1680	Q
Apl|evm.model.LG09.685	COQ5	1	Y
Apl|evm.model.LG14.527	DZIP1L	509	R
Apl|evm.model.LG21.326	CPSF11L	8	N
Apl|evm.model.LG21.407	PAXIP1	82	R

Apl|evm.model.LG01.1074	ANKEF1	338	R
Apl|evm.model.LG01.837	HNRNPLL	438	R
Apl|evm.model.LG03.644	CAT	303	A
Apl|evm.model.LG05.796	ANO8	126	R
Apl|evm.model.LG08.314	CCT5	3	N
Apl|evm.model.LG10.284	RSPH1	272	K
Apl|evm.model.LG11.545	DDX39B	163	F
Apl|evm.model.LG13.712	-	150	V
Apl|evm.model.LG15.459	-	113	R
Apl|evm.model.LG15.611	GPN2	238	R
Apl|evm.model.LG15.648	eif2s3	17	S
Apl|evm.model.LG16.14	TTLL10	147	C
Apl|evm.model.LG16.178	CEP104	3	A
Apl|evm.model.LG16.95	VEPH1	80	P
Apl|evm.model.LG01.260	ADCK1	486	M
Apl|evm.model.LG01.913	-	509	L
Apl|evm.model.LG02.1406	FBXO8	114	V
Apl|evm.model.LG02.918	KIDINS220	7	N
Apl|evm.model.LG03.693	MGAT5	69	G
Apl|evm.model.LG04.978	-	226	S
Apl|evm.model.LG06.874	PCIF1	71	Q
Apl|evm.model.LG09.272	MMAB	36	S
Apl|evm.model.LG10.173	RRP1A	252	R
Apl|evm.model.LG10.365	ETFB	149	C
Apl|evm.model.LG11.819	-	405	S
Apl|evm.model.LG13.268	RABEPK	338	T
Apl|evm.model.LG13.55	UVSSA	267	L
Apl|evm.model.LG13.649	ERI1	27	S
Apl|evm.model.LG15.178	DDX18	36	S
Apl|evm.model.LG15.456	PSPC1	477	L
Apl|evm.model.LG18.136	TRIT1	58	C
Apl|evm.model.LG18.278	ENOSF1	5	C
Apl|evm.model.LG19.273	VTI1A	117	Y
Apl|evm.model.LG01.1125	YLPM1	145	F
Apl|evm.model.LG02.1216	LRRC4	499	C
Apl|evm.model.LG04.494	NISCH	131	S
Apl|evm.model.LG04.846	DCLRE1C	415	S
Apl|evm.model.LG05.339	Tm1	283	Y
Apl|evm.model.LG07.78	PDPK1	429	S
Apl|evm.model.LG07.871	U2AF2	367	S
Apl|evm.model.LG13.49	LYAR	137	F
Apl|evm.model.LG14.542	-	2	C
Apl|evm.model.LG16.42	TMCO4	92	S

Apl|evm.model.LG16.505	MORN1	241	T
Apl|evm.model.LG17.341	GALNT11	26	H
Apl|evm.model.LG19.122	RASSF2	9	P
Apl|evm.model.LG01.1295	WDR43	9	G
Apl|evm.model.LG03.231	WAC	429	S
Apl|evm.model.LG03.513	-	144	F
Apl|evm.model.LG03.806	SP3	346	Q
Apl|evm.model.LG06.313	AKTIP	261	S
Apl|evm.model.LG09.502	SLC25A1	23	L
Apl|evm.model.LG11.589	SCYL3	524	S
Apl|evm.model.LG16.79	TNK2	227	N
Apl|evm.model.LG20.443	PIFO	73	S
Apl|evm.model.LG06.621	UGT	95	M
Apl|evm.model.LG12.310	EP4	689	P
Apl|evm.model.LG06.65	CYP3A24	194	R
Apl|evm.model.LG06.313	AKTIP	261	S
Apl|evm.model.LG03.505	EPDR1	185	Y
Apl|evm.model.LG17.40	BMP1	726	A
Apl|evm.model.LG05.417	FGFR	83	D
Apl|evm.model.LG07.386	CFAP52	347	I
Apl|evm.model.LG02.591	NEK1	685	N
Apl|evm.model.LG21.397	PLK1	60	N
Apl|evm.model.LG21.9	MAP3K	1303	G

Supplementary Table S8. Positive selective gene in *A. planci* genome.

Tissues ID	Raw reads	Clean Reads	Accession numbers
Tubefeet-3	44376882	42080090	SRR26848511
Tubefeet-2	45721828	42859466	SRR26848512
Tubefeet-1	43343954	41240454	SRR26848513
Spine-apex-3	51794362	48614510	SRR26848507
Spine-apex-2	40480980	37758626	SRR26848518
Spine-apex-1	44803982	41495470	SRR26848519
Spine_base-3	47810540	45610978	SRR26848514
Spine_base-2	47809412	44144520	SRR26848515
Spine_base-1	72069248	68822832	SRR26848516
Py-sto_3	57758368	55017402	SRR26848517
Py-sto_2	47816464	45170996	SRR26848494
Py-sto_1	40159962	37956116	SRR26848495
Hep-3	52206572	48661418	SRR26848499
Hep-2	43628340	39521740	SRR26848500
Hep-1	43626906	41186202	SRR26823233
Cen_sto_3	47479054	44063114	SRR26848496
Cen_sto_2	44529324	42111172	SRR26848497
Cen_sto_1	123239396	118333548	SRR26848498
B3	54962856	51825310	SRR26848501
B2	55271412	52375436	SRR26848502
B1	60850710	57371236	SRR26848503
Amp-3	47893320	45000944	SRR26848508
Amp-2	70489924	67439152	SRR26848509
Amp-1	41422500	38962168	SRR26848510
A3	58766512	55194754	SRR26848504
A2	57232272	53947324	SRR26848505
A1	57331096	52461232	SRR26848506

Supplementary Table S9. Transcriptome sequencing data information of four individuals with different dissected tissues

References

[1] M.J. Chaisson, G. Tesler, Mapping single molecule sequencing reads using basic local alignment with successive refinement (BLASR): application and theory, Bmc Bioinformatics 13 (2012) 238.

[2] J. Hu, J. Fan, Z. Sun, et al., NextPolish: a fast and efficient genome polishing tool for long-read assembly, Bioinformatics 36 (2020) 2253-2255.

[3] N. Servant, N. Varoquaux, B.R. Lajoie, et al., HiC-Pro: an optimized and flexible pipeline for Hi-C data processing, Genome Biol. 16 (2015) 259.

[4] S. Chen, Y. Zhou, Y. Chen, et al., Fastp: an ultra-fast all-in-one FASTQ preprocessor, Bioinformatics 34 (2018) i884-i890.

[5] B. Langmead, S.L. Salzberg, Fast gapped-read alignment with Bowtie 2, 9 (2012) 357-359.

[6] F.A. Simao, R.M. Waterhouse, P. Ioannidis, et al., BUSCO: assessing genome assembly and annotation completeness with single-copy orthologs, Bioinformatics 31 (2015) 3210-3212.

[7] H. Li, R. Durbin, Fast and accurate long-read alignment with Burrows-Wheeler transform, Bioinformatics 26 (2010) 589-595.

[8] H. Li, B. Handsaker, A. Wysoker, et al., The Sequence Alignment/Map format and SAMtools, Bioinformatics 25 (2009) 2078-2079.

[9] X. Wang, L. Wang, GMATA: An Integrated Software Package for Genome-Scale SSR Mining, Marker Development and Viewing, Front. Plant Sci. 7 (2016) 1350.

[10] G. Benson, Tandem repeats finder: a program to analyze DNA sequences, Nucleic Acids Res. 27 (1999) 573-580.

[11] J.M. Flynn, R. Hubley, C. Goubert, et al., RepeatModeler2 for automated genomic discovery of transposable element families, Proc. Natl. Acad. Sci. 117 (2020) 9451-9457.

[12] W. Bao, K.K. Kojima, O. Kohany, Repbase Update, a database of repetitive elements in eukaryotic genomes, Mob. Dna 6 (2015) 11.

[13] J. Keilwagen, M. Wenk, J.L. Erickson, et al., Using intron position conservation for homology-based gene prediction, Nucleic Acids Res. 44 (2016) e89.

[14] A. Dobin, C.A. Davis, F. Schlesinger, et al., STAR: ultrafast universal RNA-seq aligner, Bioinformatics 29 (2013) 15-21.

[15] B.J. Haas, S.L. Salzberg, W. Zhu, et al., Automated eukaryotic gene structure annotation using EVidenceModeler and the Program to Assemble Spliced Alignments, Genome Biol. 9 (2008) R7.

[16] P. Jones, D. Binns, H.Y. Chang, et al., InterProScan 5: genome-scale protein function classification, Bioinformatics 30 (2014) 1236-1240.

[17] A.M. Bolger, M. Lohse, B. Usadel, Trimmomatic: a flexible trimmer for Illumina sequence data, Bioinformatics 30 (2014) 2114-2120.

[18] B. Li, C.N. Dewey, RSEM: accurate transcript quantification from RNA-Seq data with or without a reference genome, Bmc Bioinformatics 12 (2011) 1-16.

[19] G.C. Yu, L.G. Wang, Y.Y. Han, et al., ClusterProfiler: an R Package for Comparing Biological Themes Among Gene Clusters, Omics 16 (2012) 284-287.

[20] D.M. Emms, S. Kelly, OrthoFinder: phylogenetic orthology inference for comparative genomics, Genome Biol. 20 (2019) 1-14.

[21] K. Katoh, K. Misawa, K. Kuma, et al., MAFFT: a novel method for rapid multiple sequence alignment based on fast Fourier transform, Nucleic Acids Res. 30 (2002) 3059-3066.

[22] S. Capella-Gutierrez, J.M. Silla-Martinez, T. Gabaldon, TrimAl: a tool for automated alignment trimming in large-scale phylogenetic analyses, Bioinformatics 25 (2009) 1972-1973.

[23] P. Kuck, G.C. Longo, FASconCAT-G: extensive functions for multiple sequence alignment preparations concerning phylogenetic studies, Front. Zool. 11 (2014) 81.
[24] B.Q. Minh, H.A. Schmidt, O. Chernomor, et al., IQ-TREE 2: New models and efficient methods for phylogenetic inference in the genomic era, Mol. Biol. Evol. 37 (2020) 1530-1534.
[25] T. De Bie, N. Cristianini, J.P. Demuth, et al., CAFE: a computational tool for the study of gene family evolution, Bioinformatics 22 (2006) 1269-1271.
[26] M. Kimura, A simple method for estimating evolutionary rates of base substitutions through comparative studies of nucleotide sequences, J. Mol. Evol. 16 (1980) 111-120.
[27] C.B. Albertin, S. Medina-Ruiz, T. Mitros, et al., Genome and transcriptome mechanisms driving cephalopod evolution, Nat. Commun. 13 (2022) 2427.
[28] O. Simakov, J. Bredeson, K. Berkoff, et al., Deeply conserved synteny and the evolution of metazoan chromosomes, Sci. Adv. 8 (2022) eabi5884.
[29] C. Soderlund, W. Nelson, A. Shoemaker, et al., SyMAP: A system for discovering and viewing syntenic regions of FPC maps, Genome Res. 16 (2006) 1159-1168.
[30] M. Suyama, D. Torrents, P. Bork, PAL2NAL: robust conversion of protein sequence alignments into the corresponding codon alignments, Nucleic Acids Res. 34 (2006) W609-W612.
[31] S.L. Kosakovsky Pond, A.F.Y. Poon, R. Velazquez, et al., Hyphy 2.5-a customizable platform for evolutionary hypothesis testing using phylogenies, Mol. Biol. Evol. 37 (2020) 295-299.
[32] L.G. Macías, E. Barrio, C. Toft, GWideCodeML: A python package for testing evolutionary hypotheses at the genome-wide level, G3-genes Genom. Genet. 10 (2020) 4369-4372.
[33] C.A. Ettensohn, Lessons from a gene regulatory network: echinoderm skeletogenesis provides insights into evolution, plasticity and morphogenesis, Development 136 (2009) 11-21.
[34] S.B.T. de-Leon, The evolution of biomineralization through the co-option of organic scaffold forming networks, Cells 11 (2022) 595.
[35] H. Li, R. Durbin, Inference of human population history from individual whole-genome sequences, Nature 475 (2011) 493-496.
[36] O. Simakov, T. Kawashima, F. Marlétaz, et al., Hemichordate genomes and deuterostome origins, Nature 527 (2015) 459-465.
[37] Y. Li, A. Omori, R.L. Flores, et al., Genomic insights of body plan transitions from bilateral to pentameral symmetry in Echinoderms, Commun. Biol. 3 (2020) 371.
[38] S. Kinjo, M. Kiyomoto, T. Yamamoto, et al., HpBase: A genome database of a sea urchin, *Hemicentrotus pulcherrimus*, Dev. Growth Differ. 60 (2018) 174-182.
[39] E. Sodergren, G.M. Weinstock, E.H. Davidson, et al., The genome of the sea urchin *Strongylocentrotus purpuratus*, Science 314 (2006) 941-952.
[40] Y. Lee, B. Kim, J. Jung, et al., Chromosome-level genome assembly of *Plazaster borealis* sheds light on the morphogenesis of multiarmed starfish and its regenerative capacity, GigaScience 11 (2022) giac063.
[41] P. Kudtarkar, R.A. Cameron, Echinobase: an expanding resource for echinoderm genomic information, Database 2017 (2017) bax074.
[42] J. Jung, S.Y. Jhang, B. Kim, et al., The first high-quality genome assembly and annotation of *Patiria pectinifera*, Sci. Data 10 (2023) 642.
[43] X.J. Zhang, L.N. Sun, J.B. Yuan, et al., The sea cucumber genome provides insights into morphological evolution and visceral regeneration, Plos Biology 15 (2017) e2003790.
[44] L. Zhang, J. He, P. Tan, et al., The genome of an apodid holothuroid (*Chiridota heheva*) provides

insights into its adaptation to a deep-sea reducing environment, Commun. Biol. 5 (2022) 224.
[45] T. Chen, C. Ren, N.-K. Wong, et al., The *Holothuria leucospilota* genome elucidates sacrificial organ expulsion and bioadhesive trap enriched with amyloid-patterned proteins, Proc. Natl. Acad. Sci. 120 (2023) e2213512120.
[46] E. Parey, O. Ortega-Martinez, J. Delroisse, et al., The brittle star genome illuminates the genetic basis of animal appendage regeneration, Nat. Ecol. Evol. 8.8 (2024) 1-17.
[47] C. Han, Q. Zhang, Y. Li, et al., Chromosome-level genome assembly and annotation of the cold-water species *Ophiura sarsii*, Sci. Data 11 (2024) 560.
[48] K. Howe, M.D. Clark, C.F. Torroja, et al., The zebrafish reference genome sequence and its relationship to the human genome, Nature 496 (2013) 498-503.
[49] V.A. Schneider, T. Graves-Lindsay, K. Howe, et al., Evaluation of GRCh38 and de novo haploid genome assemblies demonstrates the enduring quality of the reference assembly, Genome Res. 27 (2017) 849-864.
[50] T. Mitros, J.B. Lyons, A.M. Session, et al., A chromosome-scale genome assembly and dense genetic map for *Xenopus tropicalis*, Dev. Biol. 452 (2019) 8-20.
[51] Y. Satou, K. Mineta, M. Ogasawara, et al., Improved genome assembly and evidence-based global gene model set for the chordate *Ciona intestinalis*: new insight into intron and operon populations, Genome Biol. 9 (2008) 1-11.
[52] O. Simakov, F. Marlétaz, J.-X. Yue, et al., Deeply conserved synteny resolves early events in vertebrate evolution, Nat. Ecol. Evol. 4 (2020) 820-830.
[53] O. Simakov, F. Marletaz, S.-J. Cho, et al., Insights into bilaterian evolution from three spiralian genomes, Nature 493 (2013) 526-531.
